# Development of preimplantation genetic testing for monogenic reference materials using next-generation sequencing

**DOI:** 10.1186/s12920-024-01803-z

**Published:** 2024-01-23

**Authors:** Weihua Zhao, Yanyan Song, Chuanfeng Huang, Shan Xu, Qi Luo, Runsi Yao, Nan Sun, Bo Liang, Jia Fei, Fangfang Gao, Jie Huang, Shoufang Qu

**Affiliations:** 1 Department of Obstetrics, Shenzhen Second People’s Hospital/the First Affiliated Hospital of Shenzhen University Health, Shenzhen, Guangdong, China; 2 BGI-Shenzhen, Guangdong Shenzhen, China; 3 Division of Physical and Chemical Testing, Division of in Vitro Diagnostic Reagents, National Institutes for food and drug Control (NIFDC), Beijing, China; 4 Laboratory of Metabolic and Developmental Sciences, State Key Laboratory of Microbial Metabolism, Shanghai, China; 5 Basecare Medical Device Co., Ltd, Jiangsu, China; 6 Peking Jabrehoo Med Tech Co., Ltd, Beijing, China; 7 Yikon Genomics Company,Ltd, Jiangsu, China

**Keywords:** Preimplantation genetic testing, Reference materials, Next-generation sequencing, Thalassemia, Monogenic disorders

## Abstract

**Objective:**

Preimplantation genetic testing for monogenic disorders (PGT-M) has been used for over 20 years to detect many serious genetic conditions. However, there is still a lack of reference materials (RMs) to validate the test performance during the development and quality control of PGT-M.

**Method:**

Sixteen thalassemia cell lines from four thalassemia families were selected to establish the RMs. Each family consisted of parents with heterozygous mutations for α- and/or β-thalassemia and two children, at least one of whom carried a homozygous thalassemia mutation (proband). The RM panel consisted of 12 DNA samples (parents and probands in 4 families) and 4 simulated embryos (cell lines constructed from blood samples from the four nonproband children). Four accredited genetics laboratories that offer verification of thalassemia samples were invited to evaluate the performance of the RM panel. Furthermore, the stability of the RMs was determined by testing after freeze‒thaw cycles and long-term storage.

**Results:**

PGT-M reference materials containing 12 genome DNA (gDNA) reference materials and 4 simulated embryo reference materials for thalassemia testing were successfully established. Next-generation sequencing was performed on the samples. The genotypes and haplotypes of all 16 PGT-M reference materials were concordant across the four labs, which used various testing workflows. These well-characterized PGT-M reference materials retained their stability even after 3 years of storage.

**Conclusion:**

The establishment of PGT-M reference materials for thalassemia will help with the standardization and accuracy of PGT-M in clinical use.

**Supplementary Information:**

The online version contains supplementary material available at 10.1186/s12920-024-01803-z.

## Introduction

Thalassemia, one of the most prevalent monogenic diseases worldwide, is a hereditary hemolytic anemia caused by the reduction or absence of α- or β-globin chain synthesis [1]. Every year, at least 330,000 babies with thalassemia are born worldwide, and the carrier rate of the thalassemia gene in pregnant women is as high as 7% [2]. In southern China, the prevalence of α-thalassemia and β-thalassemia are 8.53% and 2.54%, respectively [3, 4]. Thalassemia is a complex autosomal recessive genetic disorder; α-thalassemia is mainly caused by deletions, while β-thalassemia frequently results from single-nucleotide variations (SNVs) or oligonucleotide insertion/deletions (Indels). Most fetuses with severe α-thalassemia die in utero or soon after birth, and although patients with severe forms of β-thalassemia can survive, they require regular blood transfusions and may gradually accumulate too much iron, leading to significant economic and mental burdens on their family [3].

Preimplantation genetic testing for monogenic disorders (PGT-M) has become an increasingly popular method to reduce the risk of couples having children with monogenic conditions. In vitro fertilization (IVF) combined with PGT-M is widely used to identify whether embryos carry a genetic disorder before implantation and pregnancy [5]. Since 1998, an increasing number of couples have delivered thalassemia-free babies through PGT-M [6, 7]. Several approaches have been successfully used to test for thalassemia in PGT-M, including polymerase chain reaction (PCR)-based detection [8–10] and short tandem repeat (STR) haplotyping [11, 12]. However, these approaches are labor intensive and are restricted by the adverse influence of allele dropouts (ADOs) and limited STR loci. Recently, next-generation sequencing (NGS) combined with single-nucleotide polymorphism (SNP)-based haplotyping has shown favorable results when used in PGT-M [13, 14]. The use of NGS reduces ADOs, improving the accuracy of monogenic disease detection. Although PGT-M has been widely used over the past 20 years, there is a lack of pedigree-based reference materials that can be used to evaluate the accuracy and efficiency of the technology.

Genomic reference materials (RMs) are urgently needed for the validation of PGT-M development and quality control of its performance. With the continuous development of genetic tests used in clinical and public health practice, there has been an overall trend of increased detection accuracy compared with earlier PGT-M techniques. With the standardization and rapid evolution of PGT-M, it has been recommended that DNA RMs be used in clinical genetic testing laboratories, especially for complex recessive diseases [15]. To further improve PGT-M, genomic DNA RMs used in clinical practice should be thoroughly characterized using multiple commonly used PGT-M detection methods and should contain a complete pedigree for haplotyping.

In this study, samples from four thalassemia families were used to establish PGT-M RMs, and these RMs were validated using an NGS platform combined with SNP-based haplotyping. The validation was carried out in four research institutions, and all of the results support the feasibility of these reference materials for use in PGT-M testing.

## Materials and methods

### Selection and collection of samples

This study was approved by MGI’s institutional review board on bioethics and the Ethics Committee of The Second People’s Hospital of Shenzhen. Sixteen participants from four thalassemia families were recruited (Table 1). The parents in each family carry a heterozygous (Het) α- and/or β-thalassemia mutation, and each couple has two children. In each family, the child with a homozygous (Homo) mutation of a homotypic thalassemia gene was defined as the proband, and the sample from their sibling was considered a simulated embryo. All family members with α- and/or β-thalassemia mutant genotypes signed an informed consent form and agreed to donate whole blood for B lymphoblast cell line development.

**Table 1:** Information on thalassemia families and established cell lines

Families	Relationship	Genotypes	HGVS nomenclature	Clinical severity	Cell line no.	Package	Sample type
Family 1	Husband 1	–α^3.7^/αα, Codons 41/42(-TTCT)/β^N^	NG_000006.1: g.34164_37967del, *HBB*: c.126_129delCTTT	Thalassemia trait	CNGB030011	20 ng/µL, 15 µL/tube	gDNA
	Wife 1	Gγ(Aγδβ)^0^/β^N^	NG_000007.3: g.48795_127698del	Thalassemia trait	CNGB030012	20 ng/µL, 15 µL/tube	gDNA
	Offspring 1–1 (Proband)	Codons 41/42(-TTCT)/Gγ(Aγδβ)^0^	*HBB*: c.126_129delCTTT, NG_000007.3: g.48795_127698del	Thalassemia major	CNGB030014	20 ng/µL, 15 µL/tube	gDNA
	Offspring 1–2 (Simulated embryo)	–α^3.7^/αα, Gγ(Aγδβ)^0^/β^N^	NG_000006.1: g.34164_37967del, NG_000007.3: g.48795_127698del	Thalassemia trait	CNGB030013	3–5 cells/tube	Cells
Family 2	Husband 2	--^SEA^/αα	NG_000006.1: g.26264_45564del	Thalassemia trait	CNGB030015	20 ng/µL, 15 µL/tube	gDNA
	Wife 2	α^CS^α/αα	*HBA2*: c.427T > C	Thalassemia trait	CNGB030016	20 ng/µL, 15 µL/tube	gDNA
	Offspring 2 − 1 (Proband)	--^SEA^/α^CS^α	NG_000006.1: g.26264_45564del, *HBA2*: c.427T > C	Thalassemia intermedia	CNGB030018	20 ng/µL, 15 µL/tube	gDNA
	Offspring 2–2 (Simulated embryo)	α^CS^α/αα	*HBA2*: c.427T > C	Thalassemia trait	CNGB030017	3–5 cells/tube	Cells
Family 3	Husband 3	Gγ(Aγδβ)^0^/β^N^	NG_000007.3: g.48795_127698del	Thalassemia trait	CNGB030019	20 ng/µL, 15 µL/tube	gDNA
	Wife 3	Codons 41/42(-TTCT)/β^N^	*HBB*: c.126_129delCTTT	Thalassemia trait	CNGB030020	20 ng/µL, 15 µL/tube	gDNA
	Offspring 3 − 1 (Proband)	Codons 41/42(-TTCT)/Gγ(Aγδβ)^0^	*HBB*: c.126_129delCTTT, NG_000007.3: g.48795_127698del	Thalassemia major	CNGB030328	20 ng/µL, 15 µL/tube	gDNA
	Offspring 3 − 2 (Simulated embryo)	Gγ(Aγδβ)^0^/β^N^	NG_000007.3: g.48795_127698del	Thalassemia trait	CNGB030021	3–5 cells/tube	Cells
Family 4	Husband 4	IVS-II-654 (C > T)/β^N^	*HBB*: c.316-197 C > T	Thalassemia trait	CNGB030008	20 ng/µL, 15 µL/tube	gDNA
	Wife 4	--^SEA^/αα, IVS-II-654 (C > T)/β^N^	NG_000006.1: g.26264_45564del, *HBB*: c.316-197 C > T	Thalassemia trait	CNGB030009	20 ng/µL, 15 µL/tube	gDNA
	Offspring 4 − 1 (Proband)	--^SEA^/αα, IVS-II-654 (C > T)/IVS-II-654 (C > T)	NG_000006.1: g.26264_45564del, *HBB*: c.316-197 C > T	Thalassemia intermedia	CNGB030004	20 ng/µL, 15 µL/tube	gDNA
	Offspring 4 − 2 (Simulated embryo)	IVS-II-654 (C > T)/β^N^	*HBB*: c.316-197 C > T	Thalassemia trait	CNGB030010	3–5 cells/tube	Cells

### Cell line generation and sample processing

Sixteen thalassemia cell lines, one from each of the four families, were established as previously described [16, 17]. The twelve cell lines constructed from the parents and probands in each family were then subjected to genomic DNA (gDNA) extraction. The extraction was conducted with an MGIEasy Magnetic Beads Genomic DNA Extraction Kit (MGI Tech Co., Ltd. Item No. 1000010524) according to the manufacturer’s instructions. Extracted gDNA was diluted to 20 ng/µL and packaged in 15 µL/tube. To mimic the embryos tested in PGT-M, the cell lines constructed with blood from the four nonproband children were used as simulated embryos. These four cell lines were sorted by fluorescence activated cell sorting (FACS) (BD FACSAria^™^ II cell sorter, BD Biosciences) and cryopreserved for genetic testing without DNA extraction. Each sorted cellular sample contained 3–5 cells with 4 µL 1×PBS in the tube. The thalassemia PGT-M RMs, including gDNA samples and simulated embryo samples, were stored at -80 °C and shipped on dry ice when necessary.

### Verification protocol

Four accredited genetics laboratories that offer verification of thalassemia samples were invited to participate in the study. Each laboratory received twelve gDNA samples extracted from thalassemia cell lines and four cellular samples sorted from the four simulated embryo cell lines. gDNA samples were directly subjected to targeted amplification and high-throughput sequencing for genotype identification, whereas the cellular samples had to undergo whole-genome amplification (WGA) to obtain enough DNA for genotype verification. The four laboratories genotyped each sample using their standard protocols; the methods, reagent kits and sequencing platforms are summarized in Table 2. In general, copy number variations (CNVs) and deletions (del) of hemoglobin genes were detected by both Gap-PCR and NGS, while point mutations (single-nucleotide variants, SNVs) were detected better by NGS. To further ensure the detection accuracy of simulated embryo RMs, validations by family-based linkage analysis were also used. SNP-based haplotyping was used to identify alleles linked to the mutations and decrease the ADO misdetection rate.

**Table 2:** Characterization methods in different laboratories

Laboratories	Sample type	Methods and reagents	Sequencing platform
Lab 1	Cells	WGA: MGIEasy Single Cell Whole Genome Amplification Kit (MGI)	DNBSEQ-G400RS (MGI Tech Co., Ltd. China)
	WGA products & gDNA	NGS library: MGICare Monogenic Disease Haplotype Phasing Kit (MGI); NGS	
Lab 2	Cells	WGA: REPLI-g Single Cell Kit (QIAGEN)	MiSeq (Illumina, USA)
	WGA products & gDNA	Gap-PCR; Sanger sequencing; NGS library: NEXTflex™ DNA-Seq Kit (Bioo Scientific); NGS	
Lab 3	Cells	WGA: PicoPLEX® WGA Kit (TAKARA)	Ion Torrent DA8600 (Thermo Fisher Scientific, USA)
	WGA products & gDNA	Gap-PCR, NGS library: Preimplantation Genetic Diagnosis Kit for Thalassemia (BASECARE); NGS	
Lab 4	Cells	WGA: REPLI-g Advanced DNA Single Cell Kit (QIAGEN);	NextSeq 550 (Illumina, USA)
	WGA products & gDNA	Gap-PCR; NGS library: *HBA*&*HBB* Pedigree Verification Kit (YIKON); NGS	

#### Laboratory 1 (Lab 1)

Target region capture library preparation and high-throughput sequencing were used to genotype the samples for thalassemia in Lab 1 (Clinical Laboratory of BGI, BGI-Shenzhen, China). Multiple displacement amplification (MDA)-based WGA was performed on the four cellular samples using the MGIEasy Single Cell Whole Genome Amplification Kit (MGI Tech Co., Ltd. Item No.: 1000007744). NGS libraries of the 12 gDNA samples and 4 WGA products were constructed using the MGICare Monogenic Disease Haplotype Phasing Kit (MGI Tech Co., Ltd. Item No.: 1000005287). The libraries were sequenced on the DNBSEQ-G400RS platform (MGI Tech Co., Ltd. China) using a DNBSEQ-G400RS High-throughput Sequencing Set (FCL PE100, MGI Tech Co., Ltd. Item No.: 1000012554). Genotype verification and subsequent genetic linkage analysis were performed. Genetic linkage analysis was performed on the alpha-globin (*HBA)* and beta-globin (*HBB)* genes, and the haplotypes were determined according to the detected number of SNPs near the mutation locus (+/− 1 Mb).

#### Laboratory 2 (Lab 2)

Gap-PCR combined with NGS was used to genotype the samples in Lab 2 (Jabrehoo, Pekin, China). An MDA-based WGA protocol was carried out on the simulated embryo samples using a REPLI-g Single Cell Kit (QIAGEN. Cat. No./ID: 150343). Multiplex PCR primers (Table S1) were used to amplify pathogenic genes from all gDNA samples and WGA products. The libraries were then prepared using a NEXTflex™ DNA-Seq Kit (Bioo Scientific, USA) and sequenced on the MiSeq platform (Illumina, USA) using a MiSeqDx Universal Kit v3 SBS (Illumina, Part Number: 15072805). Furthermore, multiplex PCR (PCR primers are shown in Table S2) and Sanger sequencing were used to ensure accurate detection of single-nucleotide variants (SNVs). To detect deletion mutations, Gap-PCR and gel electrophoresis were performed to further confirm the mutant types. NGS data were analyzed using a script written in the lab (02_STA_TRIM_FASTq.pl). Genetic linkage analysis was performed on the *HBA* and *HBB* genes, and haplotypes were defined as having at least 3 valid SNPs closely linked upstream or downstream of the target gene.

#### Laboratory 3 (Lab 3)

Gap-PCR combined with NGS was used to genotype the samples for thalassemia in Lab 3 (Basecare Medical, Suzhou, China). WGA was conducted on cell samples using a PicoPLEX® WGA Kit (TAKARA, Cat. # R30050). The NGS libraries of the gDNA samples and the WGA products were then prepared using a Preimplantation Genetic Diagnosis Kit for Thalassemia (Ion Torrent) (BASECARE, Suzhou, China). Libraries were sequenced on the Ion Torrent DA8600 platform (Thermo Fisher Scientific, USA). Bioinformatic analyses were performed to directly identify the genotype of each sample and construct the haplotypes to further reduce the errors caused by ADOs.

#### Laboratory 4 (Lab 4)

Lab 4 (Yikon Genomics, China) used different protocols to verify the SNV, CNV and deletion mutant genotypes. WGA was performed on the four cell samples using a REPLI-g Advanced DNA Single Cell Kit (QIAGEN. Cat. No./ID: 150365). First, Gap-PCR (primers for Gap-PCR are shown in Table S3) and gel electrophoresis were used to identify deletion mutations causing α- and β-thalassemia. Gap-PCR was carried out with the following reaction mix: 10 µL 2 × GoldStar Best Master Mix, 1 µL forward and reverse primer mix (5 µM each), 1 µL DNA template, and 8 µL H_2_O. The following program was used for amplification: 95 °C for 10 min; 35 cycles of 97 °C for 45 s, 65 °C for 90 s, 72 °C for 180 s; 72 °C for 5 min; and hold at 12 °C. Amplification products were verified on a 1% agarose gel. Then, the *HBA* and *HBB* genes were amplified by multiplex PCR (Primer *HBA*&*HBB* Mix Pool Kit, YIKON) separately and sequenced on a NextSeq 550 System (Illumina, USA). The NGS results were used to directly determine genotypes and to identify haplotypes based on the SNPs near the mutation locus.

### Stability tests

The gDNA samples extracted from cell lines were divided into five groups (each group contained 12 gDNA samples) for storage and freeze‒thaw stability tests. Four groups of samples were kept at a constant − 80 °C for 3 months, 6 months, 12 months and 3 years. The final group of samples was cycled six times between storage at room temperature (RT) and at -80 °C. The integrity of the DNA was then reevaluated by 2% agarose gel electrophoresis. The hemoglobin genotypes of these treated gDNA samples were reanalyzed by Lab 1.

For stability analysis of the four cellular samples, four tubes of each sample were randomly selected, and the resulting 4 × 4 tubes were divided into four groups, each containing the four simulated embryo samples. The four groups were kept at -80 °C for 3 months, 6 months, 12 months and 3 years. To quickly evaluate the storage stability of the cell samples, an identification system based on MDA rather than sequencing was used. First, the cell samples were amplified using an MGIEasy Single Cell Whole Genome Amplification Kit, and the amplification efficiency was confirmed by multiplex PCR (primers are shown in Table S4) of eight housekeeping genes. The amplicons of the housekeeping genes were then reevaluated by 2% agarose gel electrophoresis. The hemoglobin genotypes and genetic linkage analyses of these treated cellular samples were reanalyzed by Lab 1.

## Results

### Establishment of cell lines

Sixteen individuals from four thalassemia families containing the most common hemoglobin genotypes were recruited to construct national PGT-M RMs (Table 1). B lymphoblastoid cell lines from all sixteen participants were successfully established. The incorporated hemoglobin genotypes were as follows: --^SEA^/αα, α^CS^α/αα, -α^3.7^/αα, --^SEA^/α^CS^α, Codons 41/42 (-TTCT)/β^N^, IVS-II-654 (C > T)/β^N^, Gγ(Aγδβ)^0^/β^N^, Codons 41/42 (-TTCT)/Gγ(Aγδβ)^0^, and IVS-II-654 (C > T)/IVS-II-654 (C > T).

### Characterization of thalassemia cell line samples

The hemoglobin genotypes of the 12 gDNA samples and the 4 simulated embryo samples were independently characterized by four laboratories with different genetic testing methods. The test results are summarized in Table 3. The four laboratories showed 100% concordance across all samples, and the hemoglobin genotypes were perfectly consistent with expectations. The sixteen samples, including 12 gDNA samples and 4 simulated embryo samples, were well characterized as candidate thalassemia PGT-M RMs.

**Table 3:** Genotype verification of thalassemia PGT-M RMs

Family	No.	Family relationship	Genotypes	Lab 1	Lab 2	Lab 3	Lab 4
Family 1	1	Husband 1	–α^3.7^/αα, Codons 41/42(-TTCT)/β^N^	√	√	√	√
	2	Wife 1	Gγ(Aγδβ)^0^/β^N^	√	√	√	√
	3	Offspring 1–1 (Proband)	Codons 41/42(-TTCT)/Gγ(Aγδβ)^0^	√	√	√	√
	4	Offspring 1–2 (Simulated embryo)	–α^3.7^/αα, Gγ(Aγδβ)^0^/β^N^	√	√	√	√
Family 2	5	Husband 2	--^SEA^/αα	√	√	√	√
	6	Wife 2	α^CS^α/αα	√	√	√	√
	7	Offspring 2 − 1 (Proband)	--^SEA^/α^CS^α	√	√	√	√
	8	Offspring 2–2 (Simulated embryo)	α^CS^α/αα	√	√	√	√
Family 3	9	Husband 3	Gγ(Aγδβ)^0^/β^N^	√	√	√	√
	10	Wife 3	Codons 41/42(-TTCT)/β^N^	√	√	√	√
	11	Offspring 3 − 1 (Proband)	Codons 41/42(-TTCT)/Gγ(Aγδβ)^0^	√	√	√	√
	12	Offspring 3 − 2 (Simulated embryo)	Gγ(Aγδβ)^0^/β^N^	√	√	√	√
Family 4	13	Husband 4	IVS-II-654 (C > T)/β^N^	√	√	√	√
	14	Wife 4	--^SEA^/αα, IVS-II-654 (C > T)/β^N^	√	√	√	√
	15	Offspring 4 − 1 (Proband)	--^SEA^/αα, IVS-II-654 (C > T)/IVS-II-654 (C > T)	√	√	√	√
	16	Offspring 4 − 2 (Simulated embryo)	IVS-II-654 (C > T)/β^N^	√	√	√	√

### SNP-based haplotyping

To reduce the diagnostic errors caused by ADOs and ensure the accuracy of detecting monogenic diseases in embryos, it is vital to construct parental haplotypes for PGT-M. The four laboratories conducted genetic linkage analysis based on the NGS results to further reduce false-positive and false-negative rates caused by direct analysis of pathogenic sites. In the family-based genetic linkage analysis, the haplotypes of the husband were marked as F0 (mutant) and F1 (normal), and the haplotypes of the wife were marked as M0 (mutant) and M1 (normal). The haplotypes of the proband (Offspring N-1) were defined as M0/F0 (representing the mutant genetic haplotype from the mother and father, respectively). Haplotype phasing results are summarized in Table 4. The haplotype phasing results obtained from the four laboratories with different instruments and validation methods are completely consistent. This demonstrates the credibility of the haplotype results obtained from the constructed RMs. The haplotypes of these pedigree-based RMs are beneficial for evaluating the performance of PGT-M testing for thalassemia and help to avoid biases caused by ADOs. For more details about the haplotyping results in different labs, please see Tables S5-S11 and Figures S1-S4. All the haplotype results were in good agreement with the genotype results.

**Table 4:** Haplotypes of four families based on genetic linkage analyses

Family	Family relationship	Haplotype of HBA	Genotypes of HBA	Haplotype of HBB	Genotypes of HBB
Family 1	Husband 1	/	/	F0/F1	Codons 41/42(-TTCT)/β^N^
	Wife 1	/	/	M0/M1	Gγ(Aγδβ)^0^/β^N^
	Offspring 1–1 (Proband)	/	/	M0/F0	Codons 41/42(-TTCT)/Gγ(Aγδβ)^0^
	Offspring 1–2 (Simulated embryo)	/	/	M0/F1	Gγ(Aγδβ)^0^/β^N^
Family 2	Husband 2	F0/F1	--^SEA^/αα	/	/
	Wife 2	M0/M1	αα^CS^/αα	/	/
	Offspring 2 − 1 (Proband)	M0/F0	--^SEA^/αα^CS^	/	/
	Offspring 2–2 (Simulated embryo)	M0/F1	αα^CS^/αα	/	/
Family 3	Husband 3	/	/	F0/F1	Gγ(Aγδβ)^0^/β^N^
	Wife 3	/	/	M0/M1	Codons 41/42(-TTCT)/β^N^
	Offspring 3 − 1 (Proband)	/	/	M0/F0	Gγ(Aγδβ)^0^/ Codons 41/42(-TTCT)
	Offspring 3 − 2 (Simulated embryo)	/	/	M1/F0	Gγ(Aγδβ)^0^/β^N^
Family 4	Husband 4	/	/	F0/F1	IVS-II-654 (C > T)/β^N^
	Wife 4	/	/	M0/M1	IVS-II-654 (C > T)/β^N^
	Offspring 4 − 1 (Proband)	/	/	M0/F0	IVS-II-654 (C > T)/IVS-II-654 (C > T)
	Offspring 4 − 2 (Simulated embryo)	/	/	M1/F0	IVS-II-654 (C > T)/β^N^

### Stability tests

To evaluate the stability of our PGT-M RMs, their freeze‒thaw tolerance and long-term storage capacity were tested. The 12 gDNA samples showed no obvious degradation after six repeated freeze‒thaw cycles (Figure S5A). The cell samples from the four simulated embryo cell lines were subjected to MDA-based WGA, and the coverage of MDA was characterized by amplifying housekeeping genes. The seven or eight housekeeping gene amplicon bands in the gels (Figure S5B) indicate the feasibility of the quick quality control protocol.

Moreover, the PGT-M RMs showed good stability after cryopreservation for 3 months, 6 months, 12 months and 3 years. There was no obvious degradation of gDNA samples even after 3 years of storage (Figure S6A), and the cell samples were also correctly amplified after 3 years of cryopreservation (Figure S6B). The genotypes and haplotypes of these treated gDNA samples and cell samples were consistent with the expected results (data not shown).

## Discussion

In this study, we demonstrated the establishment, characterization, and stability of four thalassemia pedigree PGT-M RMs, which will help to ensure the accuracy of clinical PGT-M tests for thalassemia. Sixteen B lymphoblastoid cell lines derived from four thalassemia families were successfully established. The mutations that cause α- and/or β-thalassemia, such as deletion mutations --^SEA^/ and -α^3.7^/ and nondeletion mutations α^CS^α/, IVS-II-654 (C > T), 41/42(-TTCT) and Gγ(Aγδβ)^0^, are widely distributed in South China [18, 19]. To faithfully simulate real sampling in PGT-M, four cell lines derived from the nonproband offspring were sorted into 3–5 cells/tube so that these cellular samples could only be analyzed using WGA. Four accredited labs correctly identified the genotypes and haplotypes of all the RMs, and there were no discordant results among the four laboratories, which each used their own workflow. Furthermore, the twelve gDNA RMs exhibited good stability when subjected to freeze‒thaw cycles and after long-term storage, and the quick quality control system based on MDA and multiplex PCR further demonstrated the stability of the cellular RMs. It should be noted that prior to our study, there were no characterized PGT-M RMs for clinical thalassemia.

Thalassemia occurs at a high frequency in tropical and subtropical regions, such as Africa and Southeast Asia. When couples give birth to a child with thalassemia or discover that they are carriers of thalassemia, they should use PGT-M to reduce the likelihood of having (more) children with thalassemia. Although PGT-M is an extremely effective detection method for thalassemia, its use is still under discussion among specialists worldwide. To meet the challenge to PGT-M posed by low levels of input DNA, it is common to amplify material from a few cells or even a single cell using WGA [20], such as Taq DNA polymerase-PCR-based WGA [21], MDA method-based WGA [22] or MDA combined with PCR-based WGA [23]. The general workflow for PGT-M includes WGA followed by multiplex PCR [24], SNP arrays [25] or NGS [26]. Subsequent haplotyping often relies on STR markers or SNP markers to increase the resolution of PGT-M. While WGA and subtesting techniques have been substantially improved over the past few years, the technique can still introduce errors due to amplification failure, contamination or errors in sequencing, which may have a severe impact on the reliability of the diagnostic result. Thus, PGT-M RMs should be used to minimize the error rate during the design and clinical implementation of tests.

In our study, PGT-M RMs for thalassemia were well characterized through NGS combined with SNP-based haplotyping in four laboratories. This NGS-based protocol represents a truly generic method, and the residual risk may be lower than with conventional targeted amplification strategies [27, 28]. Furthermore, different NGS platforms were used by the four labs in this study, including the DNBSEQ-G400RS platform (MGI Tech Co., Ltd. China) with a state-of-the-art core technology called DNBSEQ (DNA nanoballs sequencing), the MiSeq or NextSeq 550 System (Illumina, USA) based on bridge PCR technology, and the Ion Torrent DA8600 platform (Thermo Fisher Scientific, USA) based on semiconductor sequencing theory. The well-characterized RMs constructed in our study can thus be used with many technologies. In Lab 2, the SNVs in α-thalassemia and the deletion mutations in β-thalassemia pathogenic variants were also validated using Sanger sequencing and GAP-PCR. In addition to the characterization of thalassemia PGT-M RMs, we established a rapid quality control strategy to evaluate the stability of simulated embryo RMs. This involved performing MDA followed by multiplex PCR amplification of eight housekeeping genes. Housekeeping genes have a relatively stable expression under normal and pathological conditions and are continuously expressed in almost all tissues at all stages of growth. This quality control strategy is thus suitable for the evaluation of WGA efficiency and cell sample stability.

Although PGT-M RMs of thalassemia were successfully established, there are still limitations in this study. Not all of the common variations [29] of thalassemia in China were incorporated in the final study, so it does not reflect the entire breadth of clinical patient situations. Nevertheless, the availability of these PGT-M RMs can help ensure the accuracy of PGT-M for thalassemia tests, which will help clinicians and benefit patients.

## Conclusion

The PGT-M RMs for thalassemia developed in this work cover some of the common, rare and composite thalassemia genotypes in China. Validation by either genotyping or haplotyping in different labs produced consistent results. The well-characterized gDNA and cellular RMs described in this study may help reduce the burden of neonatal thalassemia to families and society by improving PGT-M testing. The establishment of these dedicated gDNA and cellular RMs is essential for case studies, quality control and technical verification in thalassemia testing. More details about the PGT-M RM panel for thalassemia are available on the NIFDC website (https://www.nifdc.org.cn/nifdc/, lot number: 360027 − 201901).

### Electronic supplementary material

Below is the link to the electronic supplementary material.

Supplementary Material 1

## Data Availability

The original sequencing data have been deposited into the CNGB Sequence Archive (CNSA) of the China National GeneBank DataBase (CNGBdb) with accession number CNP0003298. The original sequencing data are controlled-access data; researchers must undergo verification before obtaining access to the data by completing an application and submitting a data permission request to CNGB Data Access (CDA). After the requirements are verified, they will obtain access to download the data. All other datasets generated for this study are included in the article/supplementary material.
