# Supplementary material for: Development of preimplantation genetic testing for monogenic reference materials using next-generation sequencing
Source: BMC Med Genomics. 2024 Jan 23;17:33. doi: 10.1186/s12920-024-01803-z (PMC10807056; doi:10.1186/s12920-024-01803-z)
Supplement: Supplementary file 1 — Supplementary Material 1 [file 12920_2024_1803_MOESM1_ESM.docx]

Supplementary Material

# Supplementary Tables and Figures

## Table of content

| Table S1-S2 | Multiplex PCR primers used for verification in Lab 2 |
| --- | --- |
| Table S3 | Gap PCR primers of different families used in Lab 4 |
| Table S4 | Primers for eight housekeeping genes amplification |
| Table S5-S6 | Haplotypes of four embryos were constructed based on family genetic linkage analysis in Lab 1 |
| Table S7 | Haplotypes were constructed based on family genetic linkage analysis in Lab 2 |
| Table S8-S11 | Haplotypes were constructed based on family genetic linkage analysis in Lab 3 |
| Figure S1-S4 | Family haplotype maps were drawn based on family genetic linkage analysis in Lab 4 |
| Figure S5 | Stability tests of the PGT-M RMs for thalassemia |

**1 Primers and sequences**

Table S1 Multiplex PCR primers for NGS in Lab 2

| Gene | Primer No. | Primer sequence (5’→3’) | Gene | Primer No. | Primer sequence (5’→3’) |
| --- | --- | --- | --- | --- | --- |
| *HBA* | 1 | GTGTTCACACAAGCACACTCAG | *HBA* | 2 | TAGGGGAAGAGTGAACTGAGCA |
|  |  | GCATGCCTACAGGTATGAGTGT |  |  | TACCAGGTTCCGACACTGACT |
| *HBA* | 3 | ACATGAGATTGGGGCTCTGATG | *HBA* | 4 | GAATGGGTCCTCGAATCATCGT |
|  |  | CCTACAAAAGACCAAGGGCAGA |  |  | CCATGTGACTAGGGCATTTGGA |
| *HBA* | 5 | AGACACATGCTGGAAGAGTACAC | *HBA* | 6 | ATTTCTGGGTGCTGTCACTGTC |
|  |  | TCAGATAGACTCGGCAGAGTGAT |  |  | TGGAGTTGGCTAACAGTTCAGG |
| *HBA* | 7 | TGAGTGCTGAAGAGAAGGGAGT | *HBA* | 8 | AACAGGGTCATGGTGAGGGTA |
|  |  | AGACGTCCATCACACAGACCAT |  |  | TCTGCCAGGAACCATGAGTGA |
| *HBA* | 9 | GGGAAGCAAACTAGGGTTGCT | *HBA* | 10 | TGGCCATATGACTACGACTCCA |
|  |  | ACAGGTGTGAGATTTGCCCTG |  |  | GTCAGAGGCTGCAGCTCTTTAA |
| *HBA* | 11 | CACCCAGAAAATGGACGAATGC | *HBA* | 12 | ATTGCCCTCCCAGAGACAGT |
|  |  | ATGGATGTGTGTGAGCCCATC |  |  | TGCAGTGACTCAGATGCTGTG |
| *HBA* | 13 | CACCACCTACAACCCTAAGCAA | *HBA* | 14 | GACCCTCAGTCTTCTGTTTGACTC |
|  |  | CTCTTCCCAAACTCGACACCTT |  |  | CAGAAAAGAAGAGGCTTCACTGGG |
| *HBA* | 15 | CCAGAAGCAGGATAGGACTAGAGA | *HBA* | 16 | TTCCTCTGGATAGAGGGTGCA |
|  |  | CCAAGGCTTTGAGCTCCCTTAT |  |  | TCTCCCCTTTTCTGTCTAGCCA |
| *HBA* | 17 | ACTCAGGATTGTACCCAGTCCA | *HBA* | 18 | CAGCTCAAAGCTGTGTAGAGGA |
|  |  | CACAAACCCTGGGTTTGGTCTA |  |  | CATCAGTCGCTGGAGCCTTTAT |
| *HBA* | 19 | TTCTGTAGGACCAGGTGTCACA | *HBA* | 20 | GAGGACTTAGGTGCTGCTAAAGAC |
|  |  | TGAGCTTTGACTTCTGTGACCC |  |  | CCTAACTGTCCTCTGCTTCATGG |
| *HBA* | 21 | CCAGAAACCAGCTCAGACGTT | *HBA* | 22 | GCTCTGCCAATCAGACTTGGA |
|  |  | ACTCGAGGCCTCAGGAAATGA |  |  | AAGGACAAAGGGAAGGCCTTG |
| *HBA* | 23 | CTAAGCTCCAGGGAAGATGGAAC | *HBA* | 24 | AAGAGAAGAGGCAGGATGGAGA |
|  |  | AGGAGGCCTTCCTGGAGATAG |  |  | TCTGTGCCATTAGGAAGTCGC |
| *HBA* | 25 | TTGGAGTGGAGGCAGAAAGAAG | *HBA* | 26 | AGTCAGTCCTTCCACTGAGTCA |
|  |  | CAGAACACATCCAAGTCCCACA |  |  | CTTGTGACTGGCAGCAGCTATA |
| *HBA* | 27 | GGCCATCAACAAGAGCTTTGAC | *HBA* | 28 | AGGTCACAGCTTGGCTATGAG |
|  |  | GACATAGAAGCGGAGGGGTTTC |  |  | CAGAAACACTTGTGTGGCCTG |
| *HBA* | 29 | AGCCCGCATCCACCCAA | *HBA* | 30 | ACTGCTGGGAATCAGCTCTGA |
|  |  | GTACAACGAGAACACGCGG |  |  | GTAGCACGAAAAAGAAGCCACC |
| *HBA* | 31 | TGCCTCTCAAGGGAAAAGGAAC | *HBA* | 32 | CGTTCATGCTCCGACAGCT |
|  |  | GCAACAGAACCACCAAATCCAC |  |  | AGGAGTTAGGGGTGTCATTGGA |
| *HBA* | 33 | CACGTTGAGCCTCCTGGATG | *HBA* | 34 | CTAACGGAGCTGAAAAGCCAGA |
|  |  | CTCATCCAGATGGTGGTGTGG |  |  | TGCAGGTGTCATAGCCTTGTG |
| *HBA* | 35 | CCCCAAAACCAAGGAGAAGCA | *HBA* | 36 | ACAGAGCCAGACCAGAATTGG |
|  |  | GTTCTCCCACCAGTTCCCATC |  |  | CATCATCATCTACCCGCTGTGT |
| *HBA* | 37 | CAGTACACCAGATGAGCTGCAA | *HBA* | 38 | ACAAAGCCAGCCCTGAACC |
|  |  | AGAGGTTGCGAAGGTGCTATTC |  |  | GTGAAGAACCTGCAGCAGCTA |
| *HBA* | 39 | CCTGGGATTGGTCATTTCTGGA | *HBA* | 40 | GTTGATGTGAAGCCGAACTACG |
|  |  | CTTCCCTTTAGCTTCTGCCTGA |  |  | GATCTTAAGAGATGCTCTCCTGCC |
| *HBA* | 41 | TGCACTGTCTTTCCTAGGAAGC | *HBA* | 42 | TGGAACACACCTCATGGAAAGC |
|  |  | CAGTTGCATCTCCTCTGTGTCA |  |  | ATGCCAGTGTACAAACCAGACC |
| *HBA* | 43 | TGAGTGACATGCTGTCACTGAG | *HBA* | 44 | GGGAACCCTGATGCTCACAC |
|  |  | ACCTCCCAAAGTGCTAGGACTA |  |  | GCTTGAACCCGGGAGCTG |
| *HBA* | 45 | TGACTGCTCTGCTGGTGTTG | *HBA* | 46 | CATCCATAGACATGTGGGCTGT |
|  |  | TCGTATCGCCACCGTGCT |  |  | ACAGACACTAGCAGGGTCTTTG |
| *HBA* | 47 | AAACACAGGGACAGAGAGCAAG | *HBA* | 48 | GTACCAGATGCCACTGAAGCAT |
|  |  | GCTAAAGCCTTTTCACAGCTGG |  |  | GTGTTTGCACTGAGGGTGAGT |
| *HBA* | 49 | TTCGGGGAAGGATCTGTTTTGG | *HBA* | 50 | TCCCCTTCATTGCCTACAGGTA |
|  |  | ACTGACCTTCCCACTCTTGTCA |  |  | GAAGGGTCGAACACAGTAACGT |
| *HBA* | 51 | CAGAGCATGCTGTAGCAGTGT | *HBA* | 52 | CCTAAGGTCCTAGAAGAGACCACT |
|  |  | AGAGCAACTCCATGAAGGTGC |  |  | GCTACAGTGCAGTTCAAAGCAG |
| *HBA* | 53 | ACAACATCCTGATCTGGGAGGA | *HBA* | 54 | CCCTCCCACAGTTTTACCTCTTC |
|  |  | AGGTAGGCATTTTTGTCCCCAG |  |  | GGTATTTCCTGTTCTCCCTCCAC |
| *HBA* | 55 | CCAGACTTCTCAGGGAGAAAACATC | *HBA* | 56 | CTGGCAAGAACTACACAGAAGC |
|  |  | CTGTTAACGGTTCCATAACCCTCC |  |  | TCCTGTTTTTGGAGCCATTTGG |
| *HBA* | 57 | CCAGCGCTCACTTACTTGTCT | *HBA* | 58 | TCTTCTCTGCTAAGACCCCACA |
|  |  | TCCTCCTCTGGCCCCCT |  |  | AGTCCCAGATAGCGTCTGTGAA |
| *HBA* | 59 | TGTGGTCTAGGAGACTCTCTTCAC | *HBA* | 60 | AGGAAACCCAGGAATCCAGAGA |
|  |  | CATCCAGAATGGGCAAATCCCA |  |  | CTGACCACAGAGTCTAGGAGAGAT |
| *HBA* | 61 | ATCACTTGAGCCCAGAAGTTCC | *HBA* | 62 | TTCCAAGCCAGGCTGATTTACC |
|  |  | GTCATAGCCGAGGTTCTGTGTT |  |  | ACAGGAGACTGACACCCTTTGT |
| *HBA* | 63 | TGCATGACCTCACCTTCTGTG | *HBA* | 64 | ATCTGGGCTCTGTGTTCTCAGT |
|  |  | ACTGAAACTTCTCTGGCCCAC |  |  | CGATATGGGAAGCCGGGAGA |
| *HBA* | 65 | CTCCCCGACTCCTAAGAGAGTAG | *HBA* | 66 | ATGCTCAGCGCCCAGGA |
|  |  | AACGTAACCAGCCCGGGTT |  |  | ATCCGCACCCGAAGGACC |
| *HBA* | 67 | AGGTCACTATTGTTGGCCAGTG | *HBA* | 68 | CAGACCTGTTCCTTTCCCCTTC |
|  |  | GAAGTGGGGATGGAGGGAGAAT |  |  | CGATAGGTAAAGGCCTGAGAGC |
| *HBA* | 69 | GCCGTGTCTCAATCTCTGCA | *HBA* | 70 | CGTTATCTACATCCCCCAACTGTTTT |
|  |  | TTGCTGACTGCCAGGTCAGA |  |  | CAACATGGTGAAACTGTGTCTCT |
| *HBA* | 71 | TTTTGTCCCAGGCAGAGTTTGG | *HBA* | 72 | CCCCACCTTTCACTTTTGAGCT |
|  |  | TGAGAGTAGACAGGTTGCCCAA |  |  | AAGCAAAGAGTGAGGGGCTG |
| *HBA* | 73 | ATGTGTCTGAGCTGAGGAAGCT | *HBA* | 74 | GCCCCAGCACTTCCTGATC |
|  |  | ATGGGTAAACTGGGAAGGGTGT |  |  | ACCTCTCAGGACAGGGGATG |
| *HBA* | 75 | AGGCATCCAGGGTTCTACTCA | *HBA* | 76 | TGGTGCTTCTGCTTCCTGTG |
|  |  | TGCTCTCAGAGGGTCACCAT |  |  | TCCAGAAGAAAAGCGGTGACAG |
| *HBA* | 77 | AACTAAAATCCGACAGGCACGG | *HBA* | 78 | CATGCTTTCACACACACACACA |
|  |  | GATCTCAGCTCACCTCAACCTC |  |  | CTGAGAATAGGAAGTTGTACACAGGT |
| *HBA* | 79 | CACAGTCTCACTGATTATTACTATGTTTCC | *HBA* | 80 | CGCCCGGCCTTATGTATTTATTTT |
|  |  | TAGCCAGGCGTAGTGGGG |  |  | AAGGCGGGTGGATCAGCT |
| *HBA* | 81 | CTTCCTTCCTCACCCCACATC | *HBA* | 82 | CGGGTAGAGGAGTCTGAATCTG |
|  |  | GCTAAGCCCCAAGTCATGGAC |  |  | GAGTGCGAGCCGGAGG |
| *HBA* | 83 | CAGGGCAGAGGATCACGC | *HBA* | 84 | CATCCAGGGTTCTACTCAGGG |
|  |  | GCAGGAGGAACGGCTACC |  |  | CTCTCAGAGGGTCACCGTGA |
| *HBA* | 85 | TGAGCTCCCAGCTAATTGCC | *HBA* | 86 | CTCTTCCTGCGCCTCACA |
|  |  | TCCCCAAAGCTCAATCCAGTAC |  |  | CTCAGCCTCCGGAGTAGGT |
| *HBA* | 87 | TTTCCTTCAGGCTGTGGGCA | *HBA* | 88 | ACCCTTCCCCAGAAGTCCA |
|  |  | GGTCTGGCAGAAGATCTTGCTT |  |  | AGGAAGTCTGGAGGTGTGGA |
| *HBA* | 89 | TTGAATGCTCCAGCCGGTTC | *HBA* | 90 | GCCCAGCCCCGTGCT |
|  |  | CAGATTCAGACTCCTCTACCCGG |  |  | AGTGCGAGCCGGGAGG |
| *HBA* | 91 | CAAGACCTACTTCCCGCACTTC | *HBA* | 92 | ACAAGTTCCTGGCTTCTGTGAG |
|  |  | GTGATCCTCTGCCCTGCGA |  |  | ATCCAGCTGCAGAGAGGTTCTA |
| *HBA* | 93 | TCAGATTCAATGCAGGTTTGCTG | *HBA* | 94 | CTCAGCACCCACTCAGCT |
|  |  | CGCACCCCTGATTTCATCTAGA |  |  | AAGGGTGATTCATGGGCTCAG |
| *HBA* | 95 | CAGGAAGACGGTGTCTTCGTT | *HBA* | 96 | CTGTCTCTACTAAAAAGACAAAAATTGTCCA |
|  |  | ACCTGCCTAAGCCTGGCAAA |  |  | CCAACCACCGTGCTAATTTTTAAATTAAATT |
| *HBA* | 97 | GCTACTCGGGAGGTTGAGGTA | *HBA* | 98 | AGGTGGCGTTTGGGATGATG |
|  |  | GTCAAATATTTTCAGGGTGCAGCTACAT |  |  | AGGTGTTTCTTCAGGGCAGTG |
| *HBA* | 99 | TGAGAAAACAGACTCGGGCAAG | *HBA* | 100 | CTGCACTCACCGCACCC |
|  |  | GCATGCCACCATGCCAATTT |  |  | CTGCGACCCCGCTGC |
| *HBA* | 101 | TGCAGGCGAGTGAGCCTT | *HBA* | 102 | ACTCGGAGCAGGTTAGGGAA |
|  |  | TCGAGAGAGCACGGCAAGAA |  |  | TGACCATGATTGGTTTTATGTGGAC |
| *HBA* | 103 | GTAAACAAGAACAGGACATGGCTG | *HBA* | 104 | GGGAAGAGCCAGAGAAAATATTTGC |
|  |  | GCCTCCCGGAGTGCTG |  |  | GAGTAGCTGGGAGTACAGGCA |
| *HBA* | 105 | GAAAAGAAAAAATCTGGCCCCTAGC | *HBA* | 106 | TACCCAGAGGTGCAGATCCAA |
|  |  | TGTCCACACCTAGTTACAGGAGT |  |  | TGGAGATTGCAGCGAGTTGAG |
| *HBA* | 107 | CGTCGTGCCACTGAACTGT | *HBA* | 108 | GTTTGCGCCACTGCATTCTAG |
|  |  | GACTACAGGCGCCCGCTAT |  |  | GAAGCCCTGAGTGGGTTAAGG |
| *HBA* | 109 | AATCACTGCCAGCTGTGGTGA | *HBA* | 110 | GGATGAGAATCTGAGGCTGACA |
|  |  | TGCTGGAGTGCAGTGTTGTAGT |  |  | ACAGATGGGGAGAAATGGTGAG |
| *HBA* | 111 | ATCCCTTCCTCAGCACAATGTC | *HBA* | 112 | CCTTGTGGCTTTAGAAGCAGCT |
|  |  | TGTGAAGAACCGAGTTTACCCC |  |  | CAGTTCCATCTTTGGCCCCTTT |
| *HBA* | 113 | GGATTCCATGTCTTTGGGAGCA | *HBA* | 114 | TGTGTTCAGCTAGGGGTTCAAG |
|  |  | TGCATGCCTTATTAGTCACCCG |  |  | CATGGCCATCTTGGAGCTAGTT |
| *HBA* | 115 | TCTCCCGAGATGAGGTAACACA | *HBA* | 116 | CTCAGGGTTTGGCTACAGAAGG |
|  |  | GATGATCCTGTCGATGTAGTCCTG |  |  | GTCTCGAATGCCACACGGAA |
| *HBA* | 117 | AGAGGCTCAGGACACACAGA | *HBA* | 118 | TGTGTTGCGACGAGGAGTTC |
|  |  | AGCACTACCAGGGATAGTCCTC |  |  | GTCTATCCACATGCATGCGGA |
| *HBA* | 119 | GGCAGTTTTCCTTAGTCCCTGT | *HBA* | 120 | CAGGACCATTTATGGACCCTCAG |
|  |  | GACATCGTGAGAGGGACAAAGT |  |  | TGCACTCCAGCCTGGAAAATAG |
| *HBA* | 121 | GCAGGTACTTTTGCCTCTTGTTC | *HBA* | 122 | CACTGTTGCTGAGAATCCTGTTC |
|  |  | ACCTTTCCCTGCTTACTCCAAAC |  |  | CATGGCTGCTGCAAAAGGAA |
| *HBA* | 123 | ACACAGCTGCCTCAGAAGC | *HBA* | 124 | ACAAGCTCACAGTTCTGGGAGT |
|  |  | CTTCCCTGAAGGCCCCTTAAAA |  |  | ACAGGTCACTGGTGTACAGGTT |
| *HBA* | 125 | CCAGCATCTCATTTTCCCCCAT | *HBA* | 126 | CATCACTGTTCCCTCAGGCAA |
|  |  | AGGACACGAGGTCATAGGTGG |  |  | CCAAAATGCTGTGAGCGATGG |
| *HBA* | 127 | ACACAGGCTCCCATATGAGAGA | *HBA* | 128 | CCAGTGTGTGTGCATATGGGTA |
|  |  | CATGGTCGTGTCCTCCTTTCAA |  |  | AGTCACAACCTGTGTTTCTCCC |
| *HBA* | 129 | GCACCACACACACCATAAACAC | *HBA* | 130 | CAATAGCGCCTACTTCACGGAA |
|  |  | CAGAGAAGAATGTGGGTGGACA |  |  | TTGGTCAGAAAGGAGTGAAGGC |
| *HBA* | 131 | ATGGCTGAAGTTCAGAGGGAAG | *HBA* | 132 | TTTTCCCTGGAATGCCAGCA |
|  |  | AACCTTCTCTCCTGGTGAACAC |  |  | CACTGCTGTCCTTCCTTCCA |
| *HBA* | 133 | CAGTTTTCAGCTGTGCCATTGG | *HBA* | 134 | TGTTGGGGTCAGATGCATGAG |
|  |  | TTCCCGGTCACTCTGCTTTCT |  |  | GTAGCTTACAAGGCGTGGAGT |
| *HBA* | 135 | TCCCTTTTCGTGAAGTCCCATC | *HBA* | 136 | TCTCACACCTGGTGAGACACT |
|  |  | TTCCATCCAAGCAGGAGAGTGT |  |  | CCAAAGACAGAGGCTTCACCA |
| *HBA* | 137 | TCCTCACCTAAGGAATGGGACA | *HBA* | 138 | ATGAGCCGGAGATGGACACT |
|  |  | GAAGAAGAGCGAGCACCTGTTA |  |  | GAGAGTCTGTAGGCTTTTCAGGAG |
| *HBA* | 139 | TCACCTGGGTGGTACAACCT | *HBA* | 140 | GGATCAGAAAGCTCCTACTGCT |
|  |  | ACGTCCCATGAGCACCAAAG |  |  | ACCTCCGGAGTTAGACTTCAGA |
| *HBA* | 141 | ATTCCCCACAGGTCCTTCCT | *HBA* | 142 | CCGGCTGATTTGAGAAGTGGA |
|  |  | GTCTTTAGCATGGGGGTGCT |  |  | CGTATGATTGCAGGTGGCTCT |
| *HBA* | 143 | AAGCATTGCCTGTTCCCCAA | *HBA* | 144 | GGAACAGCTTGAACACCCG |
|  |  | AGTGGCCACGGTGAGTACA |  |  | AGGACTCGGTGACTCTGTCT |
| *HBA* | 145 | TCCCAGGCCCCACGC | *HBA* | 146 | GCACCTTGTGCTTTAGGAGAAC |
|  |  | TGCTGCCTGGGTGCCT |  |  | CCATGCCCAGATGGGAAATCT |
| *HBA* | 147 | CAACAAGAGGCCGCTGGA | *HBA* | 148 | CACTGCCTTCTCTGCAGCA |
|  |  | TGTGGACTCCTCAAGCCCT |  |  | TGTGTATCTTGGTGGGTTTGGG |
| *HBA* | 149 | GCAAGACCTGGCTCAGCT | *HBA* | 150 | TTTCTTTCGGCTGTGGCCTAG |
|  |  | TCGTGGAGGCCCCGT |  |  | TCCAGCTTCTCCTGCCATCT |
| *HBA* | 151 | TTAAGAGAGCTGTCCCCACAGA | *HBA* | 152 | TCTCTGGACTTGCCAGTGGA |
|  |  | AGACTCTCCGTGCCATCGA |  |  | GCATGCTGCGCTCTCATTTC |
| *HBA* | 153 | CAGACCACGTCAGAAAGAAGCT | *HBA* | 154 | GTGCTGAGGGAAGCCCATC |
|  |  | TCCAATGCTTTGCCCTCGC |  |  | TCCAGGAAGGGCCGATGAT |
| *HBA* | 155 | TGCTCCTGAACCAGCAAAGAG | *HBA* | 156 | ACTTCTGCCCGGGACTCG |
|  |  | TCTGCTGCTCTCCTCAGCT |  |  | CCAGAGTGACTGCGCCAG |
| *HBA* | 157 | ACAGCTGGTTCTCACTGTTGTC | *HBA* | 158 | ACCCTCTGCTAAGATGCAGCTA |
|  |  | ACAGGATATGGACCCTCCACAT |  |  | AGTGAGAATCCACGGAGCAGA |
| *HBA* | 159 | AGGACCAGTTCTCATGTTCACTG | *HBA* | 160 | GGAAACAAATGTACCCGCAACG |
|  |  | TGACTTTCCTGTCCAACTTCCTG |  |  | GTGAGAATGACTGAGCGCCTTT |
| *HBA* | 161 | AGCTTCCGGCTGATGAAGATG | *HBA* | 162 | AGCAGAGCCAGGCTTTGTAG |
|  |  | GGAAGAACTCGGCCAAGTTCT |  |  | TCTGCCCTCACTGGAAGAGT |
| *HBA* | 163 | TAGTAGGACATGTTGGCCAAGG | *HBA* | 164 | CTTCGTTTCAGGAGCCTTGTG |
|  |  | CTCATCACCACATTCCCCCAT |  |  | CTGATAGCAGTAGGTGTTCAGGG |
| *HBA* | 165 | ACCGCTTTGTACGACCTTGTTC | *HBA* | 166 | GGGCCCCGCTGGCAT |
|  |  | TCAGGAGAGGCCAAGATGCTAA |  |  | TAGGCCATGAGGGCGCTG |
| *HBA* | 167 | GTCATCTTTTCCGCCATCTCCT | *HBA* | 168 | CCTGCGTGGAGCCGAG |
|  |  | AGACATCCTTGTCCCCTCCATA |  |  | CCTCGGGCAGCTGCTC |
| *HBA* | 169 | GGACATTTGTGTGGTGCAGAAC | *HBA* | 170 | ACACGGTGGCCTTGTGTC |
|  |  | GAGAATCGTGTCAGTGACACGT |  |  | GTTGGAGCGGGGCTCG |
| *HBA* | 171 | AGGCAGGGAAGAGGCCG | *HBA* | 172 | GCAATGGCTCCGAGATGGTA |
|  |  | GAGGCTGCACGACACCTG |  |  | GGTTGCTGCTAAGACACATGC |
| *HBA* | 173 | CCACTTTAAAGGCTGAGGCACT | *HBA* | 174 | ATTCCTCACTGTCGAACCCAAC |
|  |  | AGGTCCTAGGAAGCTGCGTTTA |  |  | CTGAGTGCACAGCTCGGATTTA |
| *HBA* | 175 | ATCGTGGAACAGCCTAGAAACC | *HBA* | 176 | CAAAGAAGCTGAAGTGGAGCATG |
|  |  | TTGCGCTAACCAGGAAAGCT |  |  | TCTCCCCCATAAGGGAGCTAAAT |
| *HBA* | 177 | AGTCTGTCAAATGGCCACTGAG | *HBA* | 178 | CCTGGGAGGACAGAGGTGA |
|  |  | CTGGGGTTGAAACGGGTCT |  |  | ATCCGGTGAACAGAGGTTTCAG |
| *HBA* | 179 | ACTCTGCTCTGGTTTTCTTGCA | *HBA* | 180 | CAGGGAGGAAATAAAGCCAGGTAG |
|  |  | GTGTGGGAATGCTCCTGAAACT |  |  | GATGGAGCAGCAGTGTTCAGAA |
| *HBA* | 181 | TGGACAGGAAGCACACATTCAG | *HBA* | 182 | GCTTGTGGTTGGTTCTGTTTCTC |
|  |  | TCCTGCTCTGTTCACACATCTG |  |  | TCCACAGGTTCAAAAACCCCTC |
| *HBA* | 183 | CCAAGATCCCCAGCGTGAG | *HBA* | 184 | CCCCGATGTAAACACACACACA |
|  |  | AACCAGGGCCTCCTCATGTA |  |  | ACACCTGTCCTAACACGTGGT |
| *HBA* | 185 | GGTCTGAAAACAGCGTCTTGC | *HBA* | 186 | TGACTCGAAGGTGTGACTTTGAG |
|  |  | CAGGGGGAGTGCGTGC |  |  | CATGGAAACTGGGACTTACCCAA |
| *HBA* | 187 | GACATTGCATGAACGGTCTTAGG | *HBA* | 188 | GGTGAACTTTGGCAAGACCAGA |
|  |  | CCTTTCTGGGTGGTAACGTTCT |  |  | GATGCTAGCGATGTCCAATGGT |
| *HBA* | 189 | TGCTTTCTCTGGCTGTGTAAGG | *HBA* | 190 | TGAGAACGTCAGCACAGACTTC |
|  |  | GATGCCCATCTGGCACAGATTA |  |  | CATGTCCAGCCCCAAAAGATGA |
| *HBA* | 191 | GCCTCTTCAGCTCTTCCTCCA | *HBA* | 192 | TCCACCACGTGGAATGATCTG |
|  |  | ACACTCACTCGTCAGGGAAGAA |  |  | GCAAACTGAGGCAGCTCCTAA |
| *HBA* | 193 | CCTCCACTCAGAAGAGTCTGTCT | *HBA* | 194 | GTGACTCTCAATGGTGTGCAGT |
|  |  | AGTGAGCATTGCTTGTGCCA |  |  | TGGTCTTCTCTGGTGACACACT |
| *HBA* | 195 | CAGGACACAACAGGCTTCTCA | *HBA* | 196 | TTCCCAAAGCTGCTGTCAGTC |
|  |  | GCAAGGGCAAGCTAGCATTTC |  |  | AGTGGACACTCTGCTCTGCA |
| *HBA* | 197 | AGATTTCACACAGCCAGGAGGA | *HBA* | 198 | TCTGGATGTTCGAGCTCTCCTT |
|  |  | CATCCTTTATGAGCTCGGGCAA |  |  | ACCAGGCATCATCCTCAGAGTT |
| *HBA* | 199 | GAAAACCCCCTGGGATAGAATCC | *HBA* | 200 | ACCCGACTCCTAGTCTCTGATG |
|  |  | CTTCTTCAGTCTTGCACGCCT |  |  | GCTATCACAGCTTCTAGCCTCTG |
| *HBA* | 201 | GTCTTGCTTAGGAACCTACCATCC | *HBA* | 202 | TCCTCCTGTCACATGACAGACT |
|  |  | CATGGTGATGAAGAGCCAAGGT |  |  | AGTGATGCAGAGCAGTATTGAGG |
| *HBA* | 203 | CATGGTTCACCTTCCTGTGGAA | *HBA* | 204 | CAGTGACAAAACAGGGGCTGA |
|  |  | ACTCATGCACAAAGGGACCTG |  |  | GAAGAAGATCGCGGCAGAGAT |
| *HBA* | 205 | CTCTTACTGCACAGAGTGGACAG | *HBA* | 206 | TTGTCTTGGCCCTGGAATATGG |
|  |  | CACAGACTGTAAGCCAGAGAGTC |  |  | TCCTGTCCCCGACATTTAGTCT |
| *HBA* | 207 | CCTCCAGGAAGGAGAATTTGAGTC | *HBA* | 208 | AAGCCTCTTGGACCTGTTGAG |
|  |  | TAGCAGTTTCCCTAGAGGTCAGG |  |  | GCTAGACAAAGGGTCCTGTGT |
| *HBA* | 209 | CAATGCAAGGTGAGAGCAAGGA | *HBA* | 210 | TTTCTCCAGGATCTGGGCTCTG |
|  |  | TTGGCTAAGTTGGCTCAAGCC |  |  | ACCTGTCAGGACTGGTTTGAGA |
| *HBA* | 211 | TAGCTCTCCTGTTGGCCTCT | *HBA* | 212 | CAGATCATCGTGCAGAACGTCA |
|  |  | CAGAGGAGACGCGAACTTTTTC |  |  | TCCTCTGATGACACCATGGTCA |
| *HBA* | 213 | CAGGGCTGAATAAAGGGTCTGA | *HBA* | 214 | GGGTCCATGTGCTTTTAAAACCAT |
|  |  | AGGACAAATTGATGGCCAGGAG |  |  | CCAATCTGTTAAAAGCTTAAGGATCAAATTC |
| *HBA* | 215 | AGGCATTCTACTTGTGCCAGTC | *HBA* | 216 | GGCAGTTCTTGCCAAGTCCT |
|  |  | TGACACCCTAAAAGAGGGTTGG |  |  | CAACTCCCTGTGACAGCAGAT |
| *HBA* | 217 | ACAACAGGTGTTCACGAGGATG | *HBA* | 218 | TGCTGACCATGAAGCTGAAGG |
|  |  | GACATGGGTCCCTGCTTTTCAT |  |  | CTTCAGAGAGAGTCTGTCCCTCA |
| *HBA* | 219 | TTCGGAGCTGTGGGTTGTCA | *HBA* | 220 | TCCCCTTTTTCTCCCCTTTTGG |
|  |  | TCCATTAGTTTCTGGCCCTGGA |  |  | CTCTTAAAGTGGGGGCCATAGAC |
| *HBA* | 221 | GAACACACACAAGAGGTGTCCT | *HBA* | 222 | ATTGTCCCACCAAAGGCTATGG |
|  |  | GAGAAAAGCTCGTGTTCGGAGA |  |  | CATCCGATGTCCATGGCTTTTG |
| *HBA* | 223 | TCTCAATTAAGCGCCCCCTAC | *HBA* | 224 | CACCTAGGTTTGGCTTAGCAGA |
|  |  | TTCCTTAGGTAAACCGCTGCTC |  |  | TGTCCAGGCAGCTTTGTCAT |
| *HBA* | 225 | GACCCAAAGTTAGAGTCACTGCT | *HBA* | 226 | AAACTGGGACTTTCACGGTGAG |
|  |  | GTCTAGTCCTGAGACCACACAGA |  |  | TGCTTACCCCAAAGTCGAACAG |
| *HBA* | 227 | ATGGACCTACCCACATTCCAGA | *HBA* | 228 | CCAGGATCCTGTCTTTGAAGCA |
|  |  | CAAGAAAGATGCAGGGCCAAAG |  |  | AGCAAATTGACAGGGAGTCCTC |
| *HBA* | 229 | TCCTTTTCCTTCCAGGTCCTGA | *HBA* | 230 | CCTGCAGAAGTTCCCATTCTGA |
|  |  | CTTCAACATTGCCACACCCTG |  |  | TCTGCTCCCCAGAAACAAACTG |
| *HBA* | 231 | TGGGGAACAGTTCCTACTCAGT | *HBA* | 232 | TGTGGGGAATACAAGCCCTCA |
|  |  | TCACGGGCATCTAGTATCCCTT |  |  | ACGTCCAGTTCCAGAACCTTTC |
| *HBA* | 233 | ACAGCAGTGAAGGCTAGTCTTC | *HBA* | 234 | AAGAACTGTCCAGGGTGGGTA |
|  |  | CTGATACCAGACCATGCCTTGA |  |  | AGCAGTGTCAAGACGGACTAAC |
| *HBA* | 235 | TTCCTACTGATGAGGCACCTGA | *HBA* | 236 | CAAGATGAGATTAGGGTGGAGACAC |
|  |  | GGAAGGACTGTCGCATTCAGAA |  |  | CTTCCTTGGGTCTCTTGACTTCTC |
| *HBA* | 237 | ATCTGCTGCAGACCAGAAAGG | *HBA* | 238 | AGATAACACCCTCCTCCAAGGA |
|  |  | TTCGAGACCATGCTGCTGTTC |  |  | CTGAGTGTCAGATAGAGTCACAGC |
| *HBA* | 239 | ATGATCTCCACAGCAAGGAGGA | *HBA* | 240 | AGATGCTGGAGTCAGGACCAT |
|  |  | ACCTAGGGATGTACTGTCATACCC |  |  | AAGGCACCTGAAACAGTCTCTG |
| *HBA* | 241 | AGTGCTTCATAGAGGCCCTCA | *HBA* | 242 | GGGCCCTTGTTCTCATTTCCA |
|  |  | CATGTCCAAGTGCTGTGGACA |  |  | TACCCTCACCATGACCCTGTT |
| *HBA* | 243 | ACTGTCAGAGCACTACTGTGTG | *HBA* | 244 | ATCGGTCCAGTGCATAGAGTCA |
|  |  | AGCACCTAGAGCAGTATCCTGA |  |  | CATGTCATGGTAGAGCCAAGCT |
| *HBA* | 245 | GGACAGAAAGGCATCCTAACTGAG | *HBA* | 246 | TATGTATGGGCCACTGTCCTGT |
|  |  | TCCTGGCTATTCCTTACCAGCA |  |  | TGACTGTTCCTGCTTGCATCC |
| *HBA* | 247 | GGTCACACAGCATCTGAGTCA | *HBA* | 248 | TGGGTACTTGGCACTCTCTGA |
|  |  | CTGAGTTAACAGGACTCTCCACC |  |  | CATGGTCCTGTGAGTGTGCA |
| *HBA* | 249 | TCTCAGGAGTTAGGCGAAAACAC | *HBA* | 250 | AAGCACTCACATTCCCTCACAC |
|  |  | TCTCTAGTCCTATCCTGCTTCTGG |  |  | TGACTGTGGTCCTGAACTGAGT |
| *HBA* | 251 | CTCCAAGACCCAGACACTCATC | *HBA* | 252 | TATTTGGGGAAGAGGCAGAAGG |
|  |  | TGACACTGAGCACACGTGTATC |  |  | TTGGGTGTGTGTGAGCTACATC |
| *HBA* | 253 | AGCACTCAGTCCAGAACATCTG | *HBA* | 254 | CCTGCAGACCCAGATAAACCTT |
|  |  | TGAGCCTTTGTCCTCTTGATGG |  |  | ACCTCTCAAGTCAGATCCATGC |
| *HBA* | 255 | ATGTGTGGTTAGGTTCCCCCTA | *HBA* | 256 | GCTGTCTCCTATTCGGATGCTT |
|  |  | CATCCAACCCTTGCCAGAATTC |  |  | GCTTGAGAAATAGATGCTGCGG |
| *HBA* | 257 | TAACCTCCTAGTGGATGCCCTT | *HBA* | 258 | GGCCTAAAAACGAGAGGTGCA |
|  |  | CCATTTCCACCAGTGGCAGATA |  |  | AAGGGACCCTCATACTCAGCT |
| *HBA* | 259 | GCAACCGAGGCATGTTCATG | *HBA* | 260 | CTGGCTTTGTCCACTAGTGCT |
|  |  | ACCTCCTCGCTCAAATGCTG |  |  | TTGGAAAGGCCCCTTGGATTC |
| *HBA* | 261 | AGGATGGCAAGGTAGGCAGA | *HBA* | 262 | GAAAACTGTGCCTTAGCCAGAG |
|  |  | TGTCCTCTCTGCCCTAGTACAC |  |  | TCACCTGTGCACATCTTGCT |
| *HBA* | 263 | TTGAAGGATGCGGCCTCAC | *HBA* | 264 | GCTGCGGAACTTGTCAAACAG |
|  |  | CTGAGGCTGAGCCCTCTTG |  |  | CTTCACCACCAGCTCTGTTCA |
| *HBA* | 265 | AGCTGCTCAGAAGAGTTGCAAG | *HBA* | 266 | TTCAAAGTCTTCCACCCCCTTC |
|  |  | ATCCAGGGTCCCCCATGTTAAA |  |  | CTCTTCAGCAGGCTTGCTAGAA |
| *HBA* | 267 | TGTGCAGCTGGATGAGAAGC | *HBA* | 268 | AATGAGGAGATTCTGGGGACCA |
|  |  | TCTCCCTTCCCAGGAGGGT |  |  | AGAGTGCTTACCTTTTCTGGGC |
| *HBA* | 269 | GCAAGGAGAACTTGGCAAGAAC | *HBA* | 270 | CTCACAGACATACGCTGGCAT |
|  |  | GAAGTGCCCAGGCTCTGTC |  |  | ACCAGGTGTGTTCTGCATGAG |
| *HBA* | 271 | CAAACACAACCCAAACCCCTTC | *HBA* | 272 | ACCCATCTGGGCCTCCAG |
|  |  | CTGAGGTATAAGTGTCCTCAGCAG |  |  | GGTGCATACCCGGTGCAG |
| *HBA* | 273 | TGGAACAAAGTAAGTGGTAAAATCTCAG | *HBA* | 274 | TTACTCCATCGGTCTCCACTCA |
|  |  | TGTGAACGGGTAACAGCGC |  |  | TAGGAAAGACAGTGCATGCAGG |
| *HBA* | 275 | GCCAACTAGTGCTTTTCCCATC | *HBA* | 276 | GAATGGCTGGCTTCAAACACAC |
|  |  | CATGCAGTTCTGGAAGAAGCTG |  |  | GTAGGCTGTTTGGAACCCACAT |
| *HBA* | 277 | TTAGGGTGGGAGTTTGGATGGA | *HBA* | 278 | GGTGCTGGCCAAAGAAAATGG |
|  |  | AGGAGACAAAACCTTGGACTGC |  |  | GTGAGGTGGACAGTTTCCAGTT |
| *HBA* | 279 | CCTCCCGGGTGAGGTACTG | *HBA* | 280 | TGACTACTGGTAGGCAGAACCA |
|  |  | CTCTGCAAAGGAGCCTGGG |  |  | AGGAGCTCTTGGGCAGAGATAT |
| *HBA* | 281 | AGAGATGGGGTTTCGCTATGTTG | *HBA* | 282 | CCAAGCTCCGAGTCAATGACA |
|  |  | TTGGGTGGACAAGTCTTTCTTTTTAAAA |  |  | GACTTTTGCCATGCCTGAAGTAG |
| *HBA* | 283 | ATAAATGGGTGGAGGACCCAGA | *HBA* | 284 | CCAGGGGAAGCCAAATCTACC |
|  |  | AACCGGGAGCACAGAAATTAGG |  |  | GTGTGGCCAAAAATTTGAACTGAAG |
| *HBA* | 285 | TCTGTCTCCTTACTGGCAATGC | *HBA* | 286 | CACTCAATAGCATCTAGGCCTGAG |
|  |  | TGCACCCTCCTGACCTAGATAT |  |  | AGGTAGGCACAAGTAGTCCTGT |
| *HBA* | 287 | TAAGACTCCCAGAGGCAAAAGC | *HBA* | 288 | CTGCTCGCAGTTTCAAAGAAGG |
|  |  | CATCGTGCCTAGCCAGAAAGTA |  |  | GATGTAGTGTTCCCTGGGTATTAGG |
| *HBA* | 289 | TCTACGCAGTCCTCCACTCTAC | *HBA* | 290 | CGGCCAAGTCACAGTAATTATTATGG |
|  |  | TTCGCCCCGACTCAGGTAA |  |  | CAACGTGGCCAAATGATGATTGA |
| *HBA* | 291 | GCTAAGCTCCGCGAGGC | *HBA* | 292 | CTGGGATGGAAATGAGACTGCA |
|  |  | CCACGGCGGGATGCG |  |  | AGCAGCACGATTAGGTCAGAAG |
| *HBA* | 293 | AGTGTCCAGTGGCTTGTAAGTG | *HBA* | 294 | CACTGTGGCTGTGGTAAAGGAT |
|  |  | GGAAACAAGCCCTGATGGTTTG |  |  | AAGGGCAGGTCCAGATTCTTCT |
| *HBA* | 295 | CGCTCACTCATTCCTTCTTCCA | *HBA* | 296 | TCCTGAAGCTCCTCTTGAGCTT |
|  |  | CTCAGCCTCTTTTCCTGTCTCA |  |  | TTGCCACTCCCTCAAATGTCAC |
| *HBA* | 297 | TACCCTTTGCAAGCACACGT | *HBA* | 298 | CTTCCCCGCCGACTTCAC |
|  |  | CACTATGTTCTCATCGCAGCCT |  |  | AGCTTCTCCCCTCCTTCCC |
| *HBA* | 299 | GTCTCCTGGGGTCCTTGAGT | *HBA* | 300 | CCGAGGGGAGTAAACAGATGC |
|  |  | ATGGGAGGAACCGGAGAGAG |  |  | TCCTTATAGAGCCTCCGGGC |
| *HBA* | 301 | GAGGTGGAGATGAGGGTTTTGG | *HBA* | 302 | GAGTTCACCGTGCAAATGCAA |
|  |  | AGGTGCGGGAAGTAGACCTT |  |  | TGAGAGTTCTAGGAGGCTCCTTT |
| *HBA* | 303 | GCAAAGCACTTCCCCATTCAC | *HBA* | 304 | CTTCCAGCCTGCTCAATGACA |
|  |  | AGGTTTGCCACTGAGGAAGTG |  |  | GAGAAGAGGGAACCTGGGGAA |
| *HBA* | 305 | TGGACCAGGCCTGGCA | *HBA* | 306 | GCCGCCATTCCTGGCTA |
|  |  | GCCTATCTCAAAAACAAAAACAAAGCA |  |  | GATGGGGAGAAGAGCACAGTTT |
| *HBA* | 307 | TACCATGTGGAGAGCTGGTCT | *HBA* | 308 | GGCCACCCCTAACTATTCTTACC |
|  |  | TAGCCTTGTCCTCAGGAGACA |  |  | AAGCTCTCCCTGATGTTGCC |
| *HBA* | 309 | TCCACAGTTCCTGGGTAAATGC | *HBA* | 310 | TGAGGTGGGCCTGCTCA |
|  |  | TGCTGCCTACTCGGACTTCATT |  |  | ACCTGCACTTGCATTGGGG |
| *HBA* | 311 | GCTGGCCCTACTCACACTTC | *HBA* | 312 | TCCACACTCACAGTACTGAATTGAG |
|  |  | GAAGCAGAAGCACCAGGAGG |  |  | TCAGGTGATCCTCTCGCCTC |
| *HBA* | 313 | GCCTGTAATCCTAGCTACTAGGAAG | *HBA* | 314 | GCCACAGGCTCTCTTTTTGGA |
|  |  | AGGAGACAGGAAAGAGAGACACT |  |  | AGAGCGAGACTCCGTCACAAA |
| *HBA* | 315 | ACGCCATTCTCCTCCCTCA | *HBA* | 316 | GTTTCTCCATGTTGGTCAGCC |
|  |  | CACTCTAGCCTGACGACACAG |  |  | CCCTGTGGTTGCAGAATGTAG |
| *HBA* | 317 | TGTCACAGTGAACCACGACC | *HBA* | 318 | CCACCACCAAGACCTACTTCC |
|  |  | ACCGGGAAGGAACAAACACC |  |  | GATCCTCTGCCCTGAGAGGAA |
| *HBA* | 319 | CCTCCAAATACCGTTAAGCTGGA | *HBA* | 320 | GCTGGCCCTACTCACACTTC |
|  |  | CCTTGGTCTGAGACAGGTAAACA |  |  | CAGAAGACCAGGAGGTCCCA |
| *HBA* | 321 | AGCCTAGGAACATGAGCAGC | *HBA* | 322 | AGAAACCCCATCTATACTAAAAATACAAAATCA |
|  |  | CGTGCCTGTCGGATTTTAGTTTAT |  |  | CTGTCTGCCACCCTCTTCT |
| *HBA* | 323 | CTGAAGGGTGCTGACCTGATG | *HBA* | 324 | CTTAAGTCTCACCTCCTCCAGGA |
|  |  | GAGATGTTGGGGTGATGAGGC |  |  | AGCTCTCCAAATGCTATCTGATGG |
| *HBA* | 325 | TGTCACAGTGAACCACGACCT | *HBA* | 326 | CGGCCCGGCACTCTTC |
|  |  | ACAGGCACCGGGAAGGAATAA |  |  | AGGAGCCCGGGTCGG |
| *HBA* | 327 | CAGAGGATCACGCGGGTT | *HBA* | 328 | ACAGCAGCTGGGACACAC |
|  |  | GCAAGAAGCATGGCCACC |  |  | CTTGGCACAAGAGTGCCCA |
| *HBA* | 329 | AGTCTCCGTAAAACCTCCCAGA | *HBA* | 330 | CCTGGCCCTCAACTGATAGGAA |
|  |  | CTCTCCTCTCCAGTCGCAATG |  |  | AACCAGCCCTCTGCTGTACAT |
| *HBA* | 331 | TGCTGCTTCAGTTGACTAAGCC | *HBA* | 332 | AGCCTGGCCAATATGGGGAA |
|  |  | ATGGCGCGATCTTGACTCACTA |  |  | ACCACCACATTTTGTTTACCCATTCA |
| *HBA* | 333 | GCCACAAAAAGAAATGAAGCACTG | *HBA* | 334 | TACAGCAGAGTGAGTGCTGCAT |
|  |  | CACTTAGTGTTATGCAGCCATCAC |  |  | TTCGGCATGGATTTGGCATTGG |
| *HBA* | 335 | TGAGATCAGCCTAACACGGTGA | *HBA* | 336 | GCCTCACCCACGTTCCTC |
|  |  | TCTGAATCGCCCTGGATCCAA |  |  | GGACAGCGCGTGGGG |
| *HBA* | 337 | GACCTTCCCCGTGTTTGAGT | *HBA* | 338 | ACATCACTACGGAGAGTGAAGC |
|  |  | CTCACCTACCACCCCATTCG |  |  | GTGTGTGGCATGTCTGTGTTAC |
| *HBA* | 339 | TCAGGGGCCCTGAGGAAG | *HBA* | 340 | CTGGGATTGCAGATGTAAGCC |
|  |  | AGTCTCCCCAACAGTGTGGA |  |  | TGACCTTATGATTTGCCCGCC |
| *HBA* | 341 | CATCCTGGCTAACACAGTGCA | *HBA* | 342 | GCACACTGGGGTAGGAAACG |
|  |  | AGCCAGAACCACGTTGGC |  |  | TCAGCCTCTCAAAGCACTAGGA |
| *HBA* | 343 | ACATAGTGAGACCTGTCTCCACA | *HBA* | 344 | GTGTTTTCTGGAGCACCTGGA |
|  |  | TGAGACGATGCTTGCTTTGTCA |  |  | CCTTTCCACGTTCATCGGGAT |
| *HBA* | 345 | ATGTGCCTGTGCACTCTTTCTC | *HBA* | 346 | TTTCTGTAGCCCCCGTATTTCG |
|  |  | GAACTACATCCGCAAAGACCCT |  |  | CAGTTGGCTGAAGGCTGAGAAT |
| *HBA* | 347 | TTGTCCCTGTCTCCTGTTCAGT | *HBA* | 348 | TCTGCTGCCGTCAGAGTCT |
|  |  | AGCTAACCTCCCTGTGACTTCA |  |  | AGAGGATCCAACCCCCGAAA |
| *HBA* | 349 | AGGCTGAAATGACAGGAGACAC | *HBA* | 350 | AGAATTCAGGTCCACCCAGGT |
|  |  | GAAGGGCTTTAGGCAGAGAACA |  |  | ACCTTACAGATGTCCACGGAGT |
| *HBA* | 351 | GCTGTTTGTGATGGGAACAGTC | *HBA* | 352 | TGTGGAGACACGACCTCCTT |
|  |  | CACAACTTGGACGTGTTTCTGG |  |  | ACACTATTCCCACGTCCCCT |
| *HBA* | 353 | CCCTGTACTACCTGGTGTTCAC | *HBA* | 354 | CTCCCCTTGCTTTGTTTCTTGG |
|  |  | GTGTCTTACTGGCCAGGATAGG |  |  | CTCAGCCTCAGTCCAGGTAATC |
| *HBA* | 355 | GCTGCTTGTGTTTGGAACAGAG | *HBA* | 356 | GTGTCATGCGAATAGGGGTGT |
|  |  | CAGACGGTTTCTCCTGCTCTTT |  |  | CTTGATCTCCTGACCTCCCGAT |
| *HBA* | 357 | CTACTGAGGGTCGTCAAGGAGA | *HBA* | 358 | TGACTGTCGGCCACTTACCA |
|  |  | TTCCTCTCCAGCTTCAGCCT |  |  | ATCCAGCACCACGACTCTGT |
| *HBA* | 359 | TGTCCTCTCTGGAGACAAGGAT | *HBA* | 360 | CTGTGCCCTCTGGTATTTCACT |
|  |  | TCCACCCCTAAGTCTTCACACT |  |  | TGACAATTGAGACGAGATGGGG |
| *HBA* | 361 | GGTTGGAGTTCATGTCTGACCA | *HBA* | 362 | AAGATGTTGCAACGGGTACGA |
|  |  | GTGTCCACATGTGGTTTGTTCC |  |  | CAGATGAGGGGAACAGTGTGG |
| *HBA* | 363 | TCCACTTAAACCCCCACTTCTG | *HBA* | 364 | ATAAAGCGGGTTTGGCAGCA |
|  |  | GCATCGTAGGTGGAGAGCTG |  |  | AAGCCCAGCTCAGTTATCCCT |
| *HBA* | 365 | GCCTCACCACTTCAGGAATGTAC | *HBA* | 366 | TATTACAGCCCCGCATCCTTC |
|  |  | CGTTTTCAAAGGAAGCTCAGCG |  |  | AGAGATGGGGTGTACCAGGAA |
| *HBA* | 367 | AGAGTGGCTTCTCCGAGGTT | *HBA* | 368 | AATCTCCAAGTCCCTGGAGCTA |
|  |  | CTTGCCTACCCTGGCATGAG |  |  | ACTCCGACTTCACAAGTGTGAC |
| *HBA* | 369 | TCCATCCCTCCACATGTTTGAC | *HBA* | 370 | TCCACGGGGACCAAAGACA |
|  |  | CCATCCAAAGGAGGGGTGAATA |  |  | AGGTCAAGAACCGTGTGCC |
| *HBA* | 371 | AGATGCCATCTCCGTGTAAGGT | *HBA* | 372 | CCCACCAAAGCACACACACT |
|  |  | AGGAGACCTCAGGTTAGGGACT |  |  | TCCTCGTCAAAGGACAAGCAC |
| *HBA* | 373 | CAGCTTGATGTTGGGGATGTGA | *HBA* | 374 | TGCCTCTTGAGTCACCAAAGAG |
|  |  | CATCTGTCATGTTCTGGGGCTC |  |  | GATCTTGGCAGGGATTTCCGA |
| *HBA* | 375 | GTGTGGAAAGCTAACGTGCTCT | *HBA* | 376 | GTCACACTGCAATGACCTTTGAC |
|  |  | AGGAGCCTGGAGAAAAACATCG |  |  | AGGATGATTTAGGTGCAGACAGC |
| *HBA* | 377 | ATCTTCCGGCAGATGGATGTTC | *HBA* | 378 | GTCCTGGGACACGTGGATG |
|  |  | GCATTTTCCAGCTCAAGGTCTG |  |  | CTGATCCTGCGCTCCCAG |
| *HBA* | 379 | CAATGGACTCGACAGAGGTCAC | *HBA* | 380 | GGCTGTTTGGAGGCTCACAG |
|  |  | CAGAGGTAACTGTGGGAGTTGG |  |  | TGGACGTGCTGGGGTTCA |
| *HBA* | 381 | GACACTCAGCCAGAAGGCAT | *HBA* | 382 | ACTGCCCATGGAAGAATCTGGA |
|  |  | AGCTCATCTCCCCTCCCAA |  |  | TTCCCTCACATCCTCTGCCTT |
| *HBA* | 383 | ATACGAGGTGGGCGAGGT | *HBA* | 384 | AAATGAGAGCGCAGCATGCA |
|  |  | ACAGGAGGGGCGATGGT |  |  | AGCTTCTTTCTGACGTGGTCTG |
| *HBA* | 385 | TACAGAGAGGAGACCGTTCCTT | *HBA* | 386 | ACCGGTGCTCAGAGACACA |
|  |  | CAGTTGCTCTCCCTTTCTGAGA |  |  | CTGTTTTACGGCTCAGGTCCAA |
| *HBA* | 387 | CAAGACCCCCAATCCTTCATGG | *HBA* | 388 | AGGAGGTGCGTCACCTCA |
|  |  | TGGTCATGACGGGGAAGCA |  |  | TGCTGAGTGCTGCCCTTG |
| *HBA* | 389 | ACAGGGGACCGGGAGTG | *HBA* | 390 | AGTCTCCGGCTCCTTACTAGAG |
|  |  | CCTTGTCCTTGGTGTCGGG |  |  | ACTCCTCCGCCAACACTTTC |
| *HBA* | 391 | GAAACCCCAAGCACATTCAGTC | *HBA* | 392 | GCTGGAAGAGGTAAAGTGGGAA |
|  |  | AAGCTGCAGTGAGGTGCTTT |  |  | CTCATGCCACCTTGGGAATGAT |
| *HBA* | 393 | ATCCTGTGCTCTAGGCCTCTG | *HBA* | 394 | TTTCTGCAGCAGCACGCT |
|  |  | TGAGGAGCGCTCCTGATCC |  |  | TGCCCCAGGCCTACAGATT |
| *HBA* | 395 | TCCTGCAGTGGCAGAATTCAG | *HBA* | 396 | CACACTGCTTGATGGACTCCTT |
|  |  | CTTCTGCCTGCCTTCTGGAAA |  |  | AGCATAGGACCCAGCATTCAAG |
| *HBA* | 397 | CATCTGCCCTGGCCTGTC | *HBA* | 398 | AGATTTCCTTCTGCCTGGACAC |
|  |  | TGGAGCCCGGCAGGT |  |  | ACCACTAAGATCTCTCTCTACCGG |
| *HBA* | 399 | CTAACTGCCTGTGAGGTAGCC | *HBA* | 400 | AAGGGCCCCCTGGCTT |
|  |  | TTGCAGTATCAGGAAGCCCTG |  |  | CACATTCCAGAATCTCAGCGGA |
| *HBA* | 401 | CTTTTCCACTTGAAGCTCTCCTCA | *HBA* | 402 | TCCCTGCAAACCTCCAGGAT |
|  |  | CAAGGCCACCGTGTCTCG |  |  | TCTGATGCGTTTCACCGACG |
| *HBA* | 403 | GGGCATGGCCCATGTTCC | *HBA* | 404 | TCACAATTCCATCCCGAGAACC |
|  |  | ATGGGTGAAGGCCCCTCC |  |  | TGACGAGCGCTGGATGATTT |
| *HBA* | 405 | GGCTTGCTGTTTACACCTGATC | *HBA* | 406 | GATTTGGTTTCCTGGGGAATGC |
|  |  | CACACCTGTAGCCTCAGCTATT |  |  | CTTGTACCCAAAGTGCCAGAGA |
| *HBA* | 407 | GCTATCTCCCAACATCCCAGTG | *HBA* | 408 | ATCAGAAGACACCTGTGCGGAT |
|  |  | GTCATTTAGCCCGAAGAAGCAG |  |  | GCAAAATGGCACTCATACCCCA |
| *HBA* | 409 | CCAGAAGCCGAGAAGTAACTGA | *HBA* | 410 | TACCCATCACACTGTCTCCTGAC |
|  |  | ACCAACCCGCTGGTCTG |  |  | CTGTGAACAATGTTCAGTAACTAACATGAA |
| *HBA* | 411 | TGGCAAAACTAACCGCAGAGA | *HBA* | 412 | AGTTCTTCTGTCTGTGTCCCAC |
|  |  | CGTAAACAACCACCGTGCTTT |  |  | TGGAAATGAAGTGGGGTTCCAG |
| *HBA* | 413 | CATGCAGCAGAAAATGAGTGCA | *HBA* | 414 | GTTCCTCAGGGAACTCTCATCAAG |
|  |  | AGCTTGTTGCTGTTTTGTTCCC |  |  | GTGAGTCTCCATGAAGTCACTGAG |
| *HBA* | 415 | CCAGACACCCCTAAAATGCTAGG | *HBA* | 416 | CCTGCTTGTTAAACACCGTCAC |
|  |  | TGCTGTAGGAGTGGCAGAAGA |  |  | CTCTGAACTGTGGGAATTTGCC |
| *HBA* | 417 | ACAACATGAGCCAGAAGCTACC | *HBA* | 418 | GCTGGAAAGGCTGGTTTTTAGC |
|  |  | TTCCCTGCAGGTTTCAGCG |  |  | ACAGACAGGAGGAAGTGAGGTT |
| *HBA* | 419 | TTTGCACCACCCACATCAGT | *HBA* | 420 | TATGCCCAGCTGTAAGCAGTTC |
|  |  | ACACCTTGTCCAGAAACCAGTC |  |  | TGAGCCCTTTGAATTCCTGAGG |
| *HBA* | 421 | AGCCTCCATTTGTGAAACAGGA | *HBA* | 422 | ACCAGCTATTACCGCACTACCT |
|  |  | TCTCACGTGCTGTGGAGATCT |  |  | TCTGGCTACTACATCGGTGGAA |
| *HBA* | 423 | TTACTACCTGTTGCCCGCTCT | *HBA* | 424 | TTTGTGCCAACACTGTCCTGT |
|  |  | CTAGAAACCACTTGGAGGCCAG |  |  | GCTTACCAAAAAGCCACCACTG |
| *HBA* | 425 | TCAGAACACCTCCTGGTAACCT | *HBA* | 426 | AGGTCAGGGACTGTGTTTTTCC |
|  |  | CTGTGGCGGTTTCTTTTCTGTG |  |  | TGGACTGTAGGGGAATGAAGCT |
| *HBA* | 427 | ACGAGTTCCCGTTTATTGTGTCA | *HBA* | 428 | GGTCTCAAGAGCAGACAATGCT |
|  |  | CTGAAACCAAATTGCAACTTGTGC |  |  | TCTGGTATTGTCCCTTGGACCA |
| *HBA* | 429 | CGTAATGACCCGCTTCTTGGT | *HBA* | 430 | CAAGACGATGAGGGTCCTGTTG |
|  |  | ACTCAGATGCTGCCCTCAGTA |  |  | CTGTGCCCACATACCAGTGTAG |
| *HBA* | 431 | GGTGTTTCTAGGCCAGATGTATAGG | *HBA* | 432 | TGATCCTGTTTGGTCCTTGATGG |
|  |  | CTCAAGGCAACACATTACAGCAC |  |  | ATGGAAGGCCATGGAATAGGGA |
| *HBA* | 433 | GGAAGTCTTCGTCCTTGAGCTT | *HBA* | 434 | GTGAGTCTTTGTGTCTGTACTCTAGTG |
|  |  | TCCTGTAAGCCAAGAAGCCTTC |  |  | GAGAAAAGGCTCTGTTCTCCTTAGC |
| *HBA* | 435 | GGGCCAGTTCAACTTTGTCCA | *HBA* | 436 | AAGCAAAGCTCATGGGGACAG |
|  |  | TTGCGGTCAGACACGATCTTG |  |  | CCATGAATTAGCTCCGTCGTCA |
| *HBA* | 437 | GAAGCCCACACACCAAATGTTC | *HBA* | 438 | TCCCGTCATCCTCCTTAACAGT |
|  |  | CTGTTTATGGGGTGTGGACAGT |  |  | CACAGACGCGATGTTCTTGTTG |
| *HBA* | 439 | ATCCAGATGTGACAGAGCTCGA | *HBA* | 440 | AGCAGCTTGCTGGTTCCAT |
|  |  | TGGTGGTGGTGTTCTCTATGGA |  |  | TCCTGGCTCTGACCTGGATAT |
| *HBA* | 441 | TGAAGGCCTCACCTCTTAATGC | *HBA* | 442 | TGCCAGGACTCTTTGATGGCTA |
|  |  | GTTGGAGCTTTTGGAGCTTGAG |  |  | TCAGGTCCCTAGACAGCTCCTT |
| *HBA* | 443 | GCACATCCTCCCTATGTTGGT | *HBA* | 444 | TCCTTGCTCATCCTCTGTGGAA |
|  |  | CCTAAATGTGTCCACAGGGAGA |  |  | CTCTTGAGACAGACAAGTGCCT |
| *HBA* | 445 | CTAAAACCTGGCTCTGCACTGA | *HBA* | 446 | CCAAGCACTTCCTTGAACACAG |
|  |  | ACTGCATACTCTCCCAACAGAC |  |  | GGTATCAGAAAACCCCAGGTCA |
| *HBA* | 447 | CTCTCTCTCTCTCTTCCTTCTGGA | *HBA* | 448 | AAAGCCCCATTGAGGGAGTGA |
|  |  | GGTACCATGGCTGGAAGCAATA |  |  | AGAGACCTGAACCTCAACCTGT |
| *HBA* | 449 | GGTGCCCGGTCATAAATTGTTG | *HBA* | 450 | GCCACCACTTTTGGCCCTA |
|  |  | CGTTAGTGGGAGTCTGCCAAAA |  |  | GACTCTTTGGAGCTGCACAGA |
| *HBA* | 451 | CAGGGAAGTTTTCCTGACCCAT | *HBA* | 452 | AGGACTTTGGTTGTGGTCTGTC |
|  |  | ACTCCTCCTGATACAGACCCAG |  |  | CAGAAGGATGGCATGGAAGTCA |
| *HBA* | 453 | GCTTGTCCTCCCTGACTAGGA | *HBA* | 454 | GCCTGGCGCATTTCTTCAAG |
|  |  | TCCTGGTCCACTCCTCATACC |  |  | TTCCTTGGACCCCTCCCAAT |
| *HBA* | 455 | TGTGTGCCATGCCAAATTGC | *HBA* | 456 | CTGGTCTTGAACTCCCAACCTT |
|  |  | CTTCATCAATGATCGCGTGCAG |  |  | AGCTCCTGCACAAACACTAGAC |
| *HBA* | 457 | ATAGGCAGGAAGGAGCAGGAA | *HBA* | 458 | ATTGCTGAGACCAGTGACCTTC |
|  |  | CTCAGCTCTGCCTCAGTTTCTC |  |  | AGAGGCCAGAGAAACTTCTGGA |
| *HBA* | 459 | TAAGGTCCCATTCTGAGGTCCA | *HBA* | 460 | AGGGCAACTTCTCCTTTGAGTC |
|  |  | AGGAGGAATATTCACCAAGCCG |  |  | TGGTAAGTGCAGTTCTGACGAG |
| *HBA* | 461 | CAGATCCAGGCTTATTCCTTTGAGA | *HBA* | 462 | CCTAGAGCCAAGATCCAAACGT |
|  |  | GAAGCAGTCAGAATTCAAACATTATAATCTTC |  |  | CCAACCAGAACTCATCAAAGCC |
| *HBA* | 463 | TGACAGACCTGTCTCACTGTCT | *HBA* | 464 | TTCCTCCGCAAACACTCTTACC |
|  |  | GAAGAGACAAAAAGAGGTAGGCAAAG |  |  | GTCAACGAGATGACTTGCGCTA |
| *HBA* | 465 | CTCCATTCCGCACAGAGTCTAC | *HBA* | 466 | GCCCCTGTGTACATTCAGAGTAG |
|  |  | ACAGAAGGGGTCAGATGGACA |  |  | GGAACGTGGCTCCATTATCCTT |
| *HBA* | 467 | GGCAAGTTTGGGAAACACTGAC |  |  |  |
|  |  | AAGGGTTAGAGACACCCAGTGA |  |  |  |
| *HBB* | 1 | TCTAAGGGTCCGCAATCTCTCT | *HBB* | 2 | CCTTCAGTTGCAGAGAGTGGTT |
|  |  | AAGCTGCAAAGCCAGAAGGT |  |  | TGGTCTTGGTGTTTTGTCCCTC |
| *HBB* | 3 | CAAAAGTGGACACAACCCCTGA | *HBB* | 4 | TTTGGCTGCAGCTGTAGCTT |
|  |  | GAAGGGGTTGTGCTAACACCTT |  |  | GTAGAGCATGTGTGGGTGAACA |
| *HBB* | 5 | CTTAAGGAATGGGTCGGTGACA | *HBB* | 6 | CTGTTATCAATTGCAGAGAATTTAGGGAA |
|  |  | AGTGGGCTCTTTTCTGAATCCC |  |  | GGAAAGAGATTTTATCTTGCAAAGAGGT |
| *HBB* | 7 | AACTACTGGTTTGCCAGCCTAG | *HBB* | 8 | TATTGCCAGGAGACTTGGACAG |
|  |  | CATCTTCCCATGTCACAGTCGT |  |  | CATCTTCAGCCCCAGACAATGA |
| *HBB* | 9 | GAGACCTGGTTTTTGCCTGGTA | *HBB* | 10 | TTTACTCATGCTTCGCCCACTC |
|  |  | GTGTGTGACCTGGAACTGTGAA |  |  | AGCAGGCAGCTACTAAAAGGTC |
| *HBB* | 11 | AAGAGAAGGCAGAGTACCCGAA | *HBB* | 12 | ACTGGTCATCACTCCACACCTA |
|  |  | CTCAGTGTCAGGCTGTGTTCTT |  |  | TTCCTTCTCACCACTCACTGGT |
| *HBB* | 13 | TGAGTTTATGGCCACAGGACTG | *HBB* | 14 | GTGCCGTGGGTAAAAACAGGAT |
|  |  | TTGGGGCCCAAAACAGGTT |  |  | GCAACTGGTGGCCTTATACTGT |
| *HBB* | 15 | TTTCCCTCCCTTATGACCCTGA | *HBB* | 16 | ATGTTCTCTCCCTTCCCTGGAT |
|  |  | GTTCCCTTTTCAGGCTTTGGAC |  |  | ACCTCATAGGCCTTTGTGGAAC |
| *HBB* | 17 | TAACACCCTTCCCTCTTGAAGC | *HBB* | 18 | GAGACATTCGTTGGGATCAGCA |
|  |  | CAACAAAACCCCCATAACATGGC |  |  | TGCAGGACTCTGTGCAAACTAG |
| *HBB* | 19 | CTGCCATTTGTGATGGTAGCATG | *HBB* | 20 | TATGCTGTTCGCACCAAGCA |
|  |  | CCTTGACCTACATCTCCCTGTTTC |  |  | GTTGGAGAGCACAGTGCCTAAT |
| *HBB* | 21 | GACCTTTGCTGGGACAAAACTG | *HBB* | 22 | AGTATGCCTTACCAGCCTGAGA |
|  |  | TCTCGAAGCCTTAGTTTTGGGC |  |  | CCATCAAGGATCTGTTGCCAGT |
| *HBB* | 23 | CAGGTTTTCTGATCCTGCCACT | *HBB* | 24 | TCCTAATCCCTCTGGGAAAGCA |
|  |  | TGTGACTCTCACCTTCTGACCT |  |  | CCATCTCTGCTGCCTCCATTAA |
| *HBB* | 25 | CCATTTGGAGTCACAGAGACCA | *HBB* | 26 | CCAAAGTGCTGGGAATACAAGC |
|  |  | CATCTAGGCCCTCAGATTGCTT |  |  | TACCGCAATTTGGATCCTCCAG |
| *HBB* | 27 | TGGAGAACCAGGTATTCCGACT | *HBB* | 28 | GCCAATAACTCCACTTTAGCTCCT |
|  |  | GTCACTGGAAGACAGCCATCAA |  |  | CTAGACTCTTGACACAGCAAGCT |
| *HBB* | 29 | TAACCTCACCTTCCAGACCCAA | *HBB* | 30 | GGCTTCACATTCATGGAGTCCT |
|  |  | TGTGGCATGAACAAACACATGC |  |  | TCTGTGCATGAGAGCTTCATCG |
| *HBB* | 31 | ACCTTAGGATTGAGCTGGGGAA | *HBB* | 32 | TGCAAGACCATTCCCTACTCCT |
|  |  | TAGCCAAGAACACAGGGCTTTC |  |  | TTGCCAAGTGGGCAACAGAT |
| *HBB* | 33 | GGCTGTTAGCCATACCATTGGA | *HBB* | 34 | AAATTCTGGGCAAGGGTGCT |
|  |  | TGCACTTGGCTTCTGTGTGT |  |  | ACAGGTAAAAGGGTGAACTGGG |
| *HBB* | 35 | TCCATCTTTGAGGATGGGAGGA | *HBB* | 36 | TTGCCCTATAGAGTCCCATCCA |
|  |  | GTTGCCTATGAAAAAGCCCTGG |  |  | CTCTCCTCAGCTTTATAAGGCTCC |
| *HBB* | 37 | GGGACATTTACTTACCTCCTCCAT | *HBB* | 38 | ATCCTCACTAGCAACAGGGTTG |
|  |  | CTCAATTGCTACTGCCTTGACTG |  |  | CAGTGCCAAGTCCAGCATAGTA |
| *HBB* | 39 | GCCCCAGATGTAAGAAGTGCT | *HBB* | 40 | TTCTGTGCCATTTCCCTCTGAG |
|  |  | TCTCTCCCTATTTGGGGCTGA |  |  | GAGTGGCAAAAGCTACAGGGAT |
| *HBB* | 41 | GCCTTTGTGCTGGGTATAGGAT | *HBB* | 42 | AGACCCTCAAAGAAGGCTATGC |
|  |  | GGAAGCAAGATGCTTTCTGGTG |  |  | GTCTGTCCCATTCCGATGGAAT |
| *HBB* | 43 | ATCCTGCACTCAATTTCCCCTA | *HBB* | 44 | GGTGGTTTTGGTTTTGAGAATTCATG |
|  |  | TCCTGGATTGGATCTTGGAGTAC |  |  | GTGAATGTGTGGCCCAATTCTG |
| *HBB* | 45 | AGGATGTAAGGCCAAGGGAGT | *HBB* | 46 | ACTCTACCACAAAAAGGGTACTGT |
|  |  | ACAGAGTGACCGTATGTAGCCT |  |  | CAACATTGAGGTCCAGTCTGCTA |
| *HBB* | 47 | GGGACAACATTGCACACATCAC | *HBB* | 48 | GTGTGAACCAAACCTGTGTGTG |
|  |  | TCTGCTTCCTCCCCTCTTTCTT |  |  | ATCGTGAAGCTGGCTACTGAAG |
| *HBB* | 49 | GAACAACGAGCCTTAGGTGCAT | *HBB* | 50 | CTGTGTCCTTTTTGCCACATGC |
|  |  | GGTTTGTGAAATCTGCCAGCAC |  |  | CTTCCGAGGGTGTTATGGCTTT |
| *HBB* | 51 | ACAGAGGACTCCATGAAGGACA | *HBB* | 52 | AGGGCTCTTTGAGTAGAGTCCT |
|  |  | TCCTCAATCTATGCCATGGTGC |  |  | CATCCAAAGGTCTCACCTGTCT |
| *HBB* | 53 | ACTGACCTCCCACATTCCCTT | *HBB* | 54 | GTGGGAGGAAGATAAGAGGTATGAAC |
|  |  | TGCAGGCTGCCTATCAGAAAG |  |  | CATGCCTCTTTGCACCATTCTAAAG |
| *HBB* | 55 | AGAAAAGAAGGGGAAAGAAAACATCAAG | *HBB* | 56 | GCCCAGTTTCTATTGGTCTCCT |
|  |  | TGGTCTACCCTTGGACCCAG |  |  | AGCAGGGAGGGCAGGAG |
| *HBB* | 57 | GGAGTTGGACTTCAAACCCTCA | *HBB* | 58 | TAAAGCACGGAGTGTGTGTGTG |
|  |  | AGACGCAGGAAGAGATCCATCT |  |  | CCATCAAGTGTTCCTGCTCCAA |
| *HBB* | 59 | GGCAGACAAATACACACCCATG | *HBB* | 60 | CAGCAGAATAGATTTATTATTTGTATTGCTTGC |
|  |  | TCCTCTCTTCCCTTCCCTTTCT |  |  | CTCAGCTCACTATGGGTTCATCTTTAT |
| *HBB* | 61 | ACTAAAGGCAACAGGGCTGAAA | *HBB* | 62 | CCTCACTGGATACTCTAAGACTATTGG |
|  |  | TCCTGGTTGTCTACCCATGGAC |  |  | CTCTATGATGGGAGAAGGAAACTAGC |
| *HBB* | 63 | CCTCGAGACTAAAGGCAACAGT | *HBB* | 64 | CTAAGACTATTGGTCAAGTTTGCCTTG |
|  |  | ATGGACCCAGAGGTTCTTTGAC |  |  | GGTATCTTCTATGGTGGGAGAAGAAAA |
| *HBB* | 65 | GTGTTTTAGGCATAGGTCCAGGA | *HBB* | 66 | TGAGGCCTCAGCTTTCTAGGAA |
|  |  | TCCTGCACTGAAACTGTTGCT |  |  | AAGGCTGCAGTGAGACATGATC |
| *HBB* | 67 | CTTTGCACAAAGGATACCCTGGA | *HBB* | 68 | TCTAGCCAGAGAACACCAAGGA |
|  |  | CCAAACCAGCTATCACTCTTCTGT |  |  | CATGTCACCCACCATTACTGCT |
| *HBB* | 69 | TGGGGCTCAATAAGTCTGCTTC | *HBB* | 70 | GTCTGCTTTCTGCCATGTTTGG |
|  |  | GTCTGTAGCTGCCAACATAGCT |  |  | GAGACCCCTTTTGGGAAACCAT |
| *HBB* | 71 | CTTCCCACCTGTTGTCAATCCT | *HBB* | 72 | GATGTGTGACATGCAGGTGTTG |
|  |  | GAGAACTTCCTGTTAGACGCCA |  |  | TGCATCACTCCTACTGTCTCCA |
| *HBB* | 73 | TGCATGCAGACAGTAGGAATGG | *HBB* | 74 | AGGAACCTTGTGCTCCACCTA |
|  |  | GCATCCTACTGGCCATGAGTTT |  |  | AGTGGCTGTGGTGTGTGTTTAG |
| *HBB* | 75 | AGATCGCAGCAAGTGAGTGTAG | *HBB* | 76 | CTTGAACAGGATCACAGGGTGT |
|  |  | GCTATCCGCAAGCTCAAGAGAT |  |  | CTTGGCTCAACTCTTTGTTGCC |
| *HBB* | 77 | AACACAGACATGGGAACCACAG | *HBB* | 78 | ATTGCCAGCTAAGAACTCCGTA |
|  |  | AGCTTGCCTGTGCTGATATCTC |  |  | ACCTGGCCAGCACTTTCTATAT |
| *HBB* | 79 | GTCTCCTTAGGGAGGAACTTTGAC | *HBB* | 80 | GGATTGAGCTCTAAAACCAAACCAT |
|  |  | AAGCCCCTTTGCATAATGGCA |  |  | TCTCTCCCCACTACTCTTTTGTTTT |
| *HBB* | 81 | GTGCTTCTCTGTGTTCTAAACACTG | *HBB* | 82 | GTTTGCTTCTCAAGCCCCTCT |
|  |  | GTACAGCACAACCACAAACTCATC |  |  | GACATCCTGAATTGCTGGTCCT |
| *HBB* | 83 | TCTCTCCTCACTGCACAAAAGG | *HBB* | 84 | ATTGGCCCACGTTTTCTGGT |
|  |  | CACATTAGGAACCGGGGGAAAT |  |  | GAGTCCGAGTGTCAACCACAAT |
| *HBB* | 85 | CAGTTACTTGGTCCTGTGGCTT | *HBB* | 86 | ATCCCAGACTGTGAGTCTGTGT |
|  |  | ACAGCGAAATACGTTTGCCCTA |  |  | CTAGTGCACCATGTGAGCCTTA |
| *HBB* | 87 | GGCATTTCTTGTGAATATATACTGAGCCTA | *HBB* | 88 | GTGACCACATGTCTGTAGCCAA |
|  |  | TGCATTGTGACAGTAGTTACTTGTAGT |  |  | GGTATTAAAGGCCTTCTGCCGA |
| *HBB* | 89 | GAGATTTGAGGAGAGTTAACAAAAAGCA | *HBB* | 90 | TCAGTGAGCTATGGGTGAGCTA |
|  |  | CAAGTGTAAACCTGGCAGGTCT |  |  | TCTGCCTTGCCTCTGATTTAGAC |
| *HBB* | 91 | ATGAGCCTGTCATCAGCAGATG | *HBB* | 92 | ATGTGTACCACACCCTACCTGA |
|  |  | ATGGTAGGAGGCAGTAGCAGAT |  |  | AGAGAGGAACTGGTAGAAGGCA |
| *HBB* | 93 | ATACCCAACAGAGCCCAAAGGA | *HBB* | 94 | CACCACACTTAAACATGGCTGAG |
|  |  | ACAGTGTCTGAGACACTGAGGT |  |  | TGGATTCTGTGTTTCCCAGATGG |
| *HBB* | 95 | ATCATCCCCCACACCAAATGTG | *HBB* | 96 | CCACCTCCATCATCTCCTCCT |
|  |  | GACAAACATGGGAACCACAGGT |  |  | GGTTGGTGGAGAAAGAGCCTAG |
| *HBB* | 97 | TAAGATTGGAGGGTGGCAGGTA | *HBB* | 98 | AGCATCTGAGATTAAGCCCTGTC |
|  |  | TTCCACCCACACTCTCCATACA |  |  | CTACTCGGATCTTAACAGATGGCT |
| *HBB* | 99 | CCTTCATACATACAGCACCACCA | *HBB* | 100 | CAGCTGCTTTGAAGAGACCCTA |
|  |  | AGGATGGATCAGAGTCTGGGAAT |  |  | TGTGTCAGTAGGACTTGGGGAT |
| *HBB* | 101 | GAGATAAGGGAAAATTGTAAGAAGAATCTTCTAC | *HBB* | 102 | GGGTAGTGACAGGCAGTAACCT |
|  |  | AACCCAAAGCTGCTGGGATAAG |  |  | TGGTGTATAGTAGAAAGCAAAGACCT |
| *HBB* | 103 | CACACCCAAACTTAAGGGTCCA | *HBB* | 104 | ATCACTGGCTCCTATGGACTCA |
|  |  | AGTCAGATTGGACAGTGCAGTT |  |  | TTGGAGAGAAGGATGTGCACAG |
| *HBB* | 105 | GACCCAATGGGTTGAAGGTGA | *HBB* | 106 | TTCCTCCAAATGCAGAGGGAAC |
|  |  | TTGCCATCCTCCTAACTCTGTC |  |  | GGAATGAAGGTGGAAGTGAGCA |
| *HBB* | 107 | GCTTCCAAGACTATAGCGCTCT | *HBB* | 108 | AACCTTTGGAAGGCCACTGTAG |
|  |  | ACCAGGATAGTTGGTCACCCTA |  |  | TCACAAAATCTGAGTGGGCCTC |
| *HBB* | 109 | TTGTTGGCTGCTCTGGTACTAG | *HBB* | 110 | TGGTAGGAAATGTGACCGTTGG |
|  |  | ACCACCATCACAGGCATTGT |  |  | AAGGAACCAAGCTCCAGAACAG |
| *HBB* | 111 | GTGAAGGTCCCTTAGGTCTAGAGT | *HBB* | 112 | CAAATCTCAGTAAAAGTGGCCTGAG |
|  |  | TCACATAGCAGTGAGAAGCAGC |  |  | ACTCAATGTAGGCGCTTCTTCAT |
| *HBB* | 113 | AATGGGAACTCAGCTTCCAAGG | *HBB* | 114 | CTGGCTGTGGTCCTTACTTACC |
|  |  | TTCCTCCTATCAGCCCACTCTT |  |  | GAACACAGCATCACTGTCCAGA |
| *HBB* | 115 | TTTGTCCCACTCCTTCCAGTTG | *HBB* | 116 | GCAGGGTTTTTCCTCAGTACCA |
|  |  | AGGTGGGTCAATTGAAAGAGGG |  |  | TGTGGAGAATGTGGCAAAACCT |
| *HBB* | 117 | GGCTAGAAACTGTGGCTAAAACTG | *HBB* | 118 | TTTGGTGAGGACACCTTCTTCG |
|  |  | GAAGAACCCTGAGGAAAAACGATG |  |  | CTTGCCCTCCTACCTTTGATGT |
| *HBB* | 119 | AACAGACACTTGGGGAAGTAGC | *HBB* | 120 | CAAGAAGAGCAAAACGCAGTCTC |
|  |  | GGTATTTGCTTCCACCAACAGC |  |  | CACACAACATCACACTCAGAATGTC |
| *HBB* | 121 | ATGAGAGTGCTGGTCAGAGAGT | *HBB* | 122 | CTGTTTGGGGAATGGTTCAAGC |
|  |  | TAGGGATGAAGTACTAGGTCAGGC |  |  | ATGGCAAGGCTATAGTTGGGTG |
| *HBB* | 123 | TCCTTGCTTGTGTCTACCCACT | *HBB* | 124 | AAAGGCAGAGAGCAAAGGAAGG |
|  |  | AGGTAAGGAGGTGCCCATGAAT |  |  | CACTTTCCAGGCTCAAGGCTTA |
| *HBB* | 125 | TGGCTGTGTAGTCTTCCATTGAC | *HBB* | 126 | GGCCACTTGGCTAATAAGGAGT |
|  |  | ATCCCACCTCTGGGAATCTATCC |  |  | GTTCTCAAGCAGCTATGACCCT |
| *HBB* | 127 | ATGGGAATACGAAGAACCCAGA | *HBB* | 128 | AGGTGGTACCCTCATTGTGCTA |
|  |  | TTCCAAATTGACTTGCCTTGGC |  |  | CAACCACACTGGGTTATTTGCC |
| *HBB* | 129 | GTGGTGGTCTTTTGCATGACAG | *HBB* | 130 | ATGTCTCTGAGGACTCTGCTCA |
|  |  | TAGGGAGCAGAATCCCAAGACA |  |  | AGCTGTATCCACAGTCCTCAGT |
| *HBB* | 131 | TGCAGAGAGACTTTGGCTTCAG | *HBB* | 132 | ATCCTTACCTGGACACTGGTGA |
|  |  | GACACAGCTGTACACACACCAA |  |  | GTAGCTATTGCTCCCCTGCATT |
| *HBB* | 133 | CAACTTCTCTAGGCCATGGCTT | *HBB* | 134 | GATGGCTTTCTTCAGGCCAGA |
|  |  | CAGAGGAATACGAAAGGTGAGGAG |  |  | GCTTAGTGTCTCCTCTGACCTTG |
| *HBB* | 135 | GAATAGCCAGCTCATAGCTGATCC | *HBB* | 136 | AGGATGCTATAACAGCGTCATGG |
|  |  | CTGTCTTGTCCAGAGAAAGGGAAG |  |  | CTGAAGTAGCTGCCATCATCACA |
| *HBB* | 137 | GCTACAGAGGACATTTTGCTCCT | *HBB* | 138 | AACATTTGCCAAAGGCCTAAGC |
|  |  | GATCGGCAAACATGCAGTAACTC |  |  | CTTCCAATCTTCCCCTTAAGTGCT |
| *HBB* | 139 | TTGTCCAAAGTCACCTCCTGTC | *HBB* | 140 | TTATTGGACCCAGAGCTGGGTA |
|  |  | TCAGACTTGAACCCAGGTCTCT |  |  | CAGTCACATTCGGACCCAAAGA |
| *HBB* | 141 | CCCTTACAATATGATACCCGGCAT | *HBB* | 142 | TCCACCACAGTGGTAAACATGC |
|  |  | TCTGGAGAACTAGTCTTCCAGCA |  |  | CACTGAAGGTAGTGCAGTGGTT |
| *HBB* | 143 | CGTGTGTGTGAGAACTCAAAGAG | *HBB* | 144 | ACTTTCATACTGGAGGCCCCAT |
|  |  | GAAGGTCTGACCCTGAGAAAGAA |  |  | GTTGATCCCCTACTATCTGCAGTG |
| *HBB* | 145 | GCCTTTGGGAGCAGAATTCTCT | *HBB* | 146 | TGTTGGTAACTCGAGCCACATC |
|  |  | GACACTGATGAGGATGTGGCAT |  |  | TCTCTTGCCACCAGAAACAGAG |
| *HBB* | 147 | GGAGTCTACTGTTGTTTGGAACG | *HBB* | 148 | CATACGAGTGCCAAAGAGGGAA |
|  |  | CAGTTTAAGGCCTGACCACACA |  |  | CTTGCAGACTGCAATACTAGGGA |
| *HBB* | 149 | TCCAGCCTGGATTTAACCATCC | *HBB* | 150 | CCGTCTTGGGGAAAAAGTACAAG |
|  |  | AACCTTTAAACAGAAGTTTATTTGATCCA |  |  | GAAGGAACATAAATGAGGGTGGC |
| *HBB* | 151 | GCAAACAACTGCTTAAGCAGCT | *HBB* | 152 | CTGTGCAGCTCTTTCTAGTGGT |
|  |  | AGGAAGGCAGTCCTCAGGTAAA |  |  | AGTGGCTAAGAAGACCTGGTTG |
| *HBB* | 153 | CATGGGAGTGGCAGTATTGGAA | *HBB* | 154 | ATGAAGCTCTAAGAGCCCCTTG |
|  |  | TACCAGAGAGATTGGCATCCCT |  |  | CAAGAGCTAATGAACAGGGGCT |
| *HBB* | 155 | GTAAGTGCAGCAAGAATCGCAA | *HBB* | 156 | CCATGCCACTCTCCATTAACATG |
|  |  | CGTTCCCAGTAAAGAAGTTCCCT |  |  | TGTCATCCACACTTTTAAAATATCTTGTT |
| *HBB* | 157 | AAGGGACACCTACACACAGAGA | *HBB* | 158 | GTGACTCACTGTATTGTGACATTCAC |
|  |  | TCCTTGGATTGGGTTTTGCCT |  |  | CTTCATGCGGGAGAAGATATCTTAAAC |
| *HBB* | 159 | AGAGGGTTATCCAGAGTAAGAGGTT | *HBB* | 160 | GTGATTTTGTGAGGGAGGAGTACC |
|  |  | TCCACTCTTCGAAGAAACCACAA |  |  | GTCTTCTTAGCTGGTGTTGGCT |
| *HBB* | 161 | AAAAGCACTGCACTGTGCAAG | *HBB* | 162 | GAGGCTGACTTTGAAAAGTGTACC |
|  |  | CACATGATCACAACCAGGGACA |  |  | AGTCCCCAACGCAAAGTTGATA |
| *HBB* | 163 | TCAGAGGTAAGCAGGGTTCTGA | *HBB* | 164 | GGCTTCACACTGATAAGCCAGA |
|  |  | GACACACTTTGTGTGCTGAATCC |  |  | GTCAGGGTCAGCTGATGTCAAA |
| *HBB* | 165 | CGTTGCTTAGACATTTTCCCAAGG | *HBB* | 166 | CATTAGCTGTTTGCAGCCTCAC |
|  |  | TGGAGACGGCACAAAATGGAA |  |  | CTTGAGCATCTGGATTCTGCCT |
| *HBB* | 167 | TCCCCCAGTTTAGTAGTTGGACT | *HBB* | 168 | AGAATAATCCAGCCTTATCCCAACC |
|  |  | CCAAGCTAGGCCCTTTTGCTAA |  |  | TTCCCTAATCTCTTTCTTTCAGGGC |
| *HBB* | 169 | CTTAGGGTTGCCCATAACAGCA | *HBB* | 170 | GCAAATGTAAGCAATAGATGGCTCT |
|  |  | AGGTGAACGTGGATGAAGTTGG |  |  | GTAGCAATTTGTACTGATGGTATGGG |
| *HBB* | 171 | ACAAATCAATGTGCTCTGTGCA | *HBB* | 172 | CTGCCTTTTATGCTGGTCCTGT |
|  |  | GTCACAGAGGCTTTTTGTTCCC |  |  | CAACTGCTGAAAGAGATGCGGT |
| *HBB* | 173 | GAGTTGGGGATTCAGCATGTGA | *HBB* | 174 | TCCCCTTCAAGCACTAGTCACT |
|  |  | CTGAGAAACAGACCAGCACTGT |  |  | CCTTTAATTCCAGATGGGGGCA |
| *HBB* | 175 | ATCCAGGTGCTTTGTGGCAT | *HBB* | 176 | GCCTATCCTTGAAAGCTCTGCA |
|  |  | GCAAAAGTCCAGGTCGCTTC |  |  | AGCTCTCAGCTCACTATGGGTT |
| *HBB* | 177 | CATCCAGGTGCTTTATGGCATC | *HBB* | 178 | CTTTGCCTTGTTCCGATTCAGTC |
|  |  | ACCAGGACAAGGGAGGGAA |  |  | TGAGACTAAGACGTGTCCCATCA |
| *HBB* | 179 | GTTGGCTCCTCATCTATCTGCA | *HBB* | 180 | GGAGGCAAAATGTGTGCAAGAG |
|  |  | CTTGGCCCTGTTTTTGTCACTG |  |  | TGCTGCCTGTTTCACCCAAT |
| *HBB* | 181 | ACTCTGAGATAGGTTTTCCCCTT | *HBB* | 182 | CCAGAAAGAAGTACATGGGCTCA |
|  |  | AGGAAGACCAACAATGTTGAGAG |  |  | GGTAGAACTGTGCACTGTGGTAA |
| *HBB* | 183 | AGAAATCGCTGTGGAGAGATGC | *HBB* | 184 | CTGGATTGGAAGGCATCAAACAC |
|  |  | TAGGGCCTCAACACATTTGGAG |  |  | CAGAACATCTGGATTTGACACGC |
| *HBB* | 185 | AACAAGCAGATCCAATGGGGAA | *HBB* | 186 | CCTGCTTTCAGCCTGTTTTCTTC |
|  |  | ATGCTAAAAGCCAGGGACTTGT |  |  | TGTCCCTCCGTGTTGTACTGAA |
| *HBB* | 187 | TTCTACCACTGTGCCCAAGATG | *HBB* | 188 | AATGTCAGGTGCATCCTGATCC |
|  |  | CACACTACGGAATAGCCCAACA |  |  | AGCATTCCTGGTGGCTACAATG |
| *HBB* | 189 | ACTATCACTCGAGTGGCACTTG | *HBB* | 190 | CATGCCACATTCTTGGCACATG |
|  |  | GTCACTCATCCCTTTTAGGGCA |  |  | GACACAATGTCTCTCGCACCTT |
| *HBB* | 191 | CATTTCGGGGTTGTGAAGCTTG | *HBB* | 192 | GAGGGCGCTGCATAAAATCATG |
|  |  | CACAGCTGATTCTTGCTCCTGA |  |  | GTACATGGGCTCATGCAGGATT |
| *HBB* | 193 | TCATATCATGGGGTAGGGGACA | *HBB* | 194 | ACTTTCTCTGTCTGCCCAGCTA |
|  |  | GTGATGCGCGATTCAAGACTAC |  |  | CTGTAGGAGCAGAACAGGCTTT |
| *HBB* | 195 | TTCAAGTTCCCAGACTCCACTG | *HBB* | 196 | CTCTGCCCGATGCTTATTCCAT |
|  |  | ATGCACTAACCACCACCCC |  |  | CAGAAACCAGTGGGAGGACAAA |
| *HBB* | 197 | TGGTGAATGTGCTCCCTACATC | *HBB* | 198 | TGGACAATGAGGAGCAGAGAGA |
|  |  | AGCTCTGATTACCCCTTTCAGC |  |  | AGAGACTGGGAAGGGAAGGAAA |
| *HBB* | 199 | ACAGGATATATGGCCTGGTGGT | *HBB* | 200 | GCACTTCAGACCAAAGAACTTAGG |
|  |  | GGTTGCCAAGAATGATGTGGAC |  |  | GCTTTTGTCAATATTTTAGGCTACTGTG |
| *HBB* | 201 | GTGGTGGAAGTCAAGTGTGGAT | *HBB* | 202 | GGAAAGAAGGGCAAGGAAGACA |
|  |  | GCTTGCTCCGTGTGGATTAGAT |  |  | GTCATTTCTAAATGGCACCAGCC |
| *HBB* | 203 | GCTGCCTGGGTAAGTATTTGGA | *HBB* | 204 | TAAACAGAACCCACGGAGACAC |
|  |  | GTCAGTCAAGCCCAGACATCTT |  |  | CAGTGTATCAGGCTGTTGCATTC |
| *HBB* | 205 | TAGCAGCTGTAGGGTGATTTCC | *HBB* | 206 | TGACAGACTCTCCAACCTCCAA |
|  |  | GATCCCTGAGGAATTGCCACTA |  |  | AAGCTCTCAACACCTGTGGTTC |
| *HBB* | 207 | TTCAGGATAGCTCCCTTCCCAT | *HBB* | 208 | CAAAAACAAATGGTGGGCCAGA |
|  |  | AGTGAGAGCATCCCTATGACCA |  |  | TCCTGACATTGTCACAGCCATT |
| *HBB* | 209 | GGTCAGTTGCTTGACGATCTGA | *HBB* | 210 | AGGAACGCAGTCTTCTGATTGG |
|  |  | AGCTACTAAGGATCTGGCTGCT |  |  | CAGATGGGCAAAAATCTGTGCC |
| *HBB* | 211 | GTGCCCACTCTCCTTTTAAAGCT | *HBB* | 212 | GCAAAAGTGCTTGTGAGAGATGG |
|  |  | CAGATAAATGTCCCTAGTCCCCATG |  |  | GTTCTGGGGCTTACCAATGATCA |
| *HBB* | 213 | CCTGTACTGTCTGTTTCCTGCT | *HBB* | 214 | ACTCAGGAACTGAGCCTAGAGT |
|  |  | TCTCACCATAGGTCCTTGTCCT |  |  | TGCTTTTTAACAGGCCTCCCA |
| *HBB* | 215 | ATAGCTTGTGCCCATGTGCA | *HBB* | 216 | TCATTTAATATCCAACAAAATCCCACCA |
|  |  | GCATGCATTGTGTTGGCAGT |  |  | AGGTAGTCACAAGGACTATCCCA |
| *HBB* | 217 | TTTCACAGAAGAGCCTCCCTTC | *HBB* | 218 | AAGAGAGCCAGACGAGATGGT |
|  |  | CTGACTCCTGCCCTTCTCATTT |  |  | AGGAGGTTGGAGAGTTAGCTGT |
| *HBB* | 219 | CCAAAGGCCATGCTCTTTTCAC | *HBB* | 220 | GGCTCTGTACCTTCTGCAATCC |
|  |  | GCTTGCTGAGGGATTGAAGTCT |  |  | AAGGAGCTGCCAGGATCAGT |
| *HBB* | 221 | GGCTGGAAGCTTTGAGTTTCCA | *HBB* | 222 | TTCTGCCCCTTCTCCATATCCT |
|  |  | TGTGAGTGGAATTGCCTAAGCC |  |  | ACACCCAGTGCCTGACTCTATA |
| *HBB* | 223 | CTCTTGTTCTTTTGCCGGTTCC | *HBB* | 224 | CCTTCAGATCTCAGCTGAAGCA |
|  |  | AGAGGTATGGCTGGGATGACTT |  |  | GCTACAGTGGTGAACAAACCAG |
| *HBB* | 225 | CCTCTCTATTGCTGAGTTTACTAAGGT | *HBB* | 226 | ACCTAGTGTGGCCTGTATCCAT |
|  |  | TGGACAAGCTTGGTATAGCCAC |  |  | GCTTCAGACTGAAGGACGAACA |
| *HBB* | 227 | AAGGAGGAGAAGGTTCCAGGAA | *HBB* | 228 | ATCTTTCCTCCTTGGTGCCTTC |
|  |  | TTGGCCTGGGATCCAATTAGTG |  |  | ACCTCCCATGCTTACTTCCTCT |

Table S2. Multiplex PCR primers for Sanger sequencing in Lab 2.

| Mutation sites | Primers for amplification | Amplicon length (bp) | Sample No. |
| --- | --- | --- | --- |
| *HBB*: CD41-42 | G10-2F2/ G10-2R2 | 517 | CNGB030011  CNGB030014  CNGB030020  CNGB030028  CNGB030013  CNGB030021 |
| *HBA*: CS | Tha-1Fb/ Tha-1Rb | 313 | CNGB030016  CNGB030018  CNGB030017 |
| *HBB*: 654 | G10-2F/ G10-2R | 722 | CNGB030009  CNGB030008  CNGB030006  CNGB030032  CNGB030004  CNGB030010 |
| *HBA*: SEA | α-THA-COM-F/α-THA-Normal-R/α-THA-SEA-R | >4000 | CNGB030010  CNGB030017 |

Table S3. Gap PCR primers of different families used in Lab 4.

| **Family** | **Primer No.** | **Primer sequence** (5’→3’) |
| --- | --- | --- |
| **Family 1** | 1 | GCTGGACACATATAAAATGCTGC |
|  |  | AGAAATTGCCTCATGTCTCT |
|  | 2 | GCTGGACACATATAAAATGCTGC |
|  |  | TGCAGGTAGTTGTTCCCCTTCA |
|  | 3 | CCCCTCGCCAAGTCCACCC |
|  |  | AAAGCACTCTAGGGTCCAGCG |
|  | 4 | CCTTGTCTCCTCTGTCCTTTC |
|  |  | GGAGGTAGGCAGTCCTCTAA |
|  | 5 | GGGAAATGAGAAGATCCAACG |
|  |  | TGCAGGTAGTTGTTCCCCTTCA |
| **Family 2** | 1 | GGGCTCTGTGTTCTCAGTATT |
|  |  | TGCTTTGTCACCCATGCT |
|  | 2 | GTCTCACCTCAATCATCCTGTG |
|  |  | CACCTCTGGGTAGGTTCTGTA |
|  | 3 | GGGAAATGAGAAGATCCAACG |
|  |  | GGGATGGTACTGAGGAGAAA |
|  | 4 | AATAAACAGAGGCCCGAACC |
|  |  | GCTCTGGGCCAACGAATTA |
| **Family 3** | 1 | GCTGGACACATATAAAATGCTGC |
|  |  | AGAAATTGCCTCATGTCTCT |
|  | 2 | GCTGGACACATATAAAATGCTGC |
|  |  | TGCAGGTAGTTGTTCCCCTTCA |
| **Family 4** | 1 | GGGCTCTGTGTTCTCAGTATT |
|  |  | TGCTTTGTCACCCATGCT |
|  | 2 | GTCTCACCTCAATCATCCTGTG |
|  |  | CACCTCTGGGTAGGTTCTGTA |
|  | 3 | GGGAAATGAGAAGATCCAACG |
|  |  | GGGATGGTACTGAGGAGAAA |
|  | 4 | AATAAACAGAGGCCCGAACC |
|  |  | GCTCTGGGCCAACGAATTA |

Table S4. Primers for eight housekeeping genes amplification.

| **Primer Name** | **Housekeeping Genes** | **Primer sequence** (5’→3’) | **Amplicons size (bp)** |
| --- | --- | --- | --- |
| Chr18-1 | *CYB5A* | GGCAACGCTTAGACTCTGTGTG | 998 |
| Chr18-2 |  | CTGCCCTTGGCCTAACTAACCT |  |
| Chr12-1 | *PRPH* | GTTCCTCAAGAAGCTGCACGAG | 744 |
| Chr12-2 |  | CGTTAGACTCTGGATCTGGCGT |  |
| Chr16-1 | *GABARAPL2* | CCAGCCAATTCATGAGTCGGTG | 595 |
| Chr16-2 |  | CCTGACAACTCGCAAGTAGCAC |  |
| Chr17-1 | *ACTG1* | GCTCAATGGGGTACTTCAGGGT | 485 |
| Chr17-2 |  | GTGGACGTTACGTAAAAGGCCC |  |
| Chr19-1 | *NDUFA7* | TGCTCTGGATGTGAAGATGCCA | 405 |
| Chr19-2 |  | TTCCAGGTAAATCCAGCCCAGG |  |
| Chr3-1 | *UQCRC1* | CAGCCAGTCAGCATCATCCAAC | 315 |
| Chr3-2 |  | GAAAGCCGGATTGCGGTAACAT |  |
| Chr8-1 | *MYC* | GGATAGCTCTGCAAGGGGAGAG | 214 |
| Chr8-2 |  | TCGTCGCAGTAGAAATACGGCT |  |
| Chr22-1 | *MIF* | AGAAGTCAGGCACGTAGCTCAG | 113 |
| Chr22-2 |  | GGCACGTTGGTGTTTACGATGA |  |

**2 Haplotype phasing and genetic linkage analysis**

**2.1 Lab 1**

Table S5* The haplotype construction of four embryos based on nuclear family genetic analysis in Lab1.

|  |  |  | **Haplotypes of father** | | | | | | **Haplotypes of mother** | | | | | |  |  |  |
| --- | --- | --- | --- | --- | --- | --- | --- | --- | --- | --- | --- | --- | --- | --- | --- | --- | --- |
| **Family** | **Gene** | **Embryo** | **F0-U** | **F0-I** | **F0-D** | **F1-U** | **F1-I** | **F1-D** | **M0-U** | **M0-I** | **M0-D** | **M1-U** | **M1-I** | **M1-D** | **Haplotypes from father** | **Haplotypes from mother** | **Genotypes of embryos** |
| Family 1 | *HBB* | Valid SNPs | 36 | 38 | 49 | 22 | 47 | 43 | 70 | 30 | 36 | 24 | 38 | 97 | / | / | / |
|  |  | Offspring 1-2 | 1 | 10 | 1 | 21 | 46 | 42 | 70 | 29 | 36 | 5 | 0 | 2 | F1 | M0 | Gγ(Aγδβ)^0^/β^N^ |
| Family 2 | *HBA* | Valid SNPs | 23 | 0 | 100 | 18 | 0 | 136 | 56 | 0 | 110 | 42 | 0 | 84 | / | / | / |
|  |  | Offspring 2-2 | 1 | 0 | 5 | 16 | 0 | 135 | 56 | 0 | 106 | 3 | 0 | 1 | F1 | M0 | α^CS^α/αα |
| Family 3 | *HBB* | Valid SNPs | 32 | 19 | 68 | 131 | 9 | 47 | 100 | 33 | 37 | 55 | 121 | 78 | / | / | / |
|  |  | Offspring 3-2 | 32 | 19 | 68 | 6 | 5 | 1 | 4 | 2 | 2 | 55 | 118 | 78 | F0 | M1 | Gγ(Aγδβ)^0^/β^N^ |
| Family 4 | *HBB* | Valid SNPs | 41 | 8 | 89 | 49 | 19 | 53 | 37 | 155 | 93 | 85 | 105 | 17 | / | / | / |
|  |  | Offspring 4-2 | 41 | 7 | 89 | 0 | 1 | 0 | 1 | 0 | 0 | 85 | 99 | 17 | F0 | M1 | IVS-II-654 (C>T)/β^N^ |

* M0 represents mutant type and M1 represents normal haplotypes of wife; F0 represents mutant type and F1 represents normal haplotypes of husband. U, I and D of each haplotype represent the upstream, internal and downstream of the target gene. And the number of SNPs detected in each region were shown in the table. The classification of haplotypes belonging to each offspring is displayed with a purple background.

Table S6* Haplotype construction of offspring 4 based on genetic analysis without proband in Lab1.

|  |  | **Haplotypes from father** | | | | | | **Haplotypes from mother** | | | | | |  |  |  |
| --- | --- | --- | --- | --- | --- | --- | --- | --- | --- | --- | --- | --- | --- | --- | --- | --- |
| **Gene** | **Embryo** | **F0-U** | **F0-I** | **F0-D** | **F1-U** | **F1-I** | **F1-D** | **M0-U** | **M0-I** | **M0-D** | **M1-U** | **M1-I** | **M1-D** | **Haplotypes from father** | **Haplotypes from mother** | **Genotypes of embryos** |
| *HBB*† | Valid SNPs | 25 | 10 | 75 | 16 | 1 | 22 | 34 | 78 | 59 | 32 | 28 | 1 | / | / | / |
|  | Offspring 4-1 | 25 | 7 | 75 | 0 | 0 | 0 | 34 | 76 | 58 | 0 | 0 | 0 | F0 | M0 | IVS-II-654 (C>T)/ IVS-II-654 (C>T) |
|  | Offspring 4-2 | 25 | 8 | 75 | 0 | 0 | 0 | 0 | 1 | 1 | 32 | 28 | 1 | F0 | M1 | IVS-II-654 (C>T)/β^N^ |

* M0 represents mutant type and M1 represents normal haplotypes of wife; F0 represents mutant type and F1 represents normal haplotypes of husband. U, I and D of each haplotype represent the upstream, internal and downstream of the target gene. And the number of SNPs detected in each region were shown in the table. The classification of haplotypes belonging to each offspring is displayed with a purple background.

**2.2 Lab 2**

Table S7* Haplotype of four families based on genetic linkage analysis.

| Family | **Family relationship** | **Haplotype of *HBA*** | **Genotypes of *HBA*** | **Haplotype of *HBB*** | **Genotypes of *HBB*** |
| --- | --- | --- | --- | --- | --- |
| Family 1 | Husband 1 | / | / | F0/F1 | Codons 41/42(-TTCT)/β^N^ |
|  | Wife 1 | / | / | M0/M1 | Gγ(Aγδβ)^0^/β^N^ |
|  | Offspring 1-1 (Proband) | / | / | M0/F0 | Codons 41/42(-TTCT)/ Gγ(Aγδβ)^0^ |
|  | Offspring 1-2 (Simulated embryo) | / | / | M0/F1 | Gγ(Aγδβ)^0^/β^N^ |
| Family 2 | Husband 2 | F0/F1 | --^SEA^/αα | / | / |
|  | Wife 2 | M0/M1 | αα^CS^/αα | / | / |
|  | Offspring 2-1 (Proband) | M0/F0 | --^SEA^/αα^CS^ | / | / |
|  | Offspring 2-2 (Simulated embryo) | M0/F1 | αα^CS^/αα | / | / |
| Family 3 | Husband 3 | / | / | F0/F1 | Gγ(Aγδβ)^0^/β^N^ |
|  | Wife 3 | / | / | M0/M1 | Codons 41/42(-TTCT)/β^N^ |
|  | Offspring 3-1 (Proband) | / | / | M0/F0 | Gγ(Aγδβ)^0^/ Codons 41/42(-TTCT) |
|  | Offspring 3-2 (Simulated embryo) | / | / | M1/F0 | Gγ(Aγδβ)^0^/β^N^ |
| Family 4 | Husband 4 | / | / | F0/F1 | IVS-II-654 (C>T)/β^N^ |
|  | Wife 4 | / | / | M0/M1 | IVS-II-654 (C>T)/β^N^ |
|  | Offspring 4-1 (Proband) | / | / | M0/F0 | IVS-II-654 (C>T)/IVS-II-654 (C>T) |
|  | Offspring 4-2 (Simulated embryo) | / | / | M1/F0 | IVS-II-654 (C>T)/β^N^ |

* M0 represents mutant type and M1 represents normal haplotypes of wife; F0 represents mutant type and F1 represents normal haplotypes of husband.

**2.3 Lab 3**

Table S8 Haplotype of *HBB* gene in family 1. *

| **Chromosome No.** | **SNP site** | **Genotype of Wife 1** | | **Genotype of Husband 1** | | **Genotype of Offspring 1-1** | | **Genotype of Offspring 1-2** | |
| --- | --- | --- | --- | --- | --- | --- | --- | --- | --- |
| / | / | M0 | M1 | F0 | F1 | M0 | F0 | M0 | F1 |
| chr11 | 4912747 | G | G | G | A | G | G | G | A |
| chr11 | 4937040 | T | G | T | T | T | T | T | T |
| chr11 | 4937319 | T | C | T | T | T | T | T | T |
| chr11 | 4949352 | CA | C | C | C | CA | C | CA | C |
| chr11 | 4964627 | C | A | A | A | C | A | C | A |
| chr11 | 4992100 | T | G | T | G | T | T | T | G |
| chr11 | 5026200 | T | C | C | C | T | C | T | C |
| chr11 | 5117647 | T | T | T | C | T | T | T | C |
| chr11 | 5192095 | del | G | A | A | del | A | del | A |
| chr11 | 5194503 |  | A | T | T |  | T |  | T |
| chr11 | 5194530 |  | T | A | A |  | A |  | A |
| chr11 | 5211198 |  | C | T | T |  | T |  | T |
| chr11 | 5211300 |  | G | A | A |  | A |  | A |
| chr11 | 5214597 |  | TTCATC | TTCATC | T |  | TTCATC |  | T |
| chr11 | 5214621 |  | T | C | T |  | C |  | T |
| chr11 | 5214669 |  | AA | TT | TT |  | TT |  | TT |
| chr11 | 5220001 |  | T | T | C |  | T |  | C |
| chr11 | 5221645 |  | C | C | G |  | C |  | G |
| chr11 | 5221825 |  | G | G | A |  | G |  | A |
| chr11 | 5222379 |  | G | G | A |  | G |  | A |
| chr11 | 5232573 |  | A | A | G |  | A |  | G |
| chr11 | 5236851 |  | C | C | T |  | C |  | T |
| chr11 | 5247791 |  | C | C | G |  | C |  | G |
| chr11 | 5247992 |  | CAAAG | **C** | CAAAG |  | **C** |  | CAAAG |
| chr11 | 5248243 |  | A | A | G |  | A |  | G |
| chr11 | 5291414 | C | T | T | T | C | T | C | T |
| chr11 | 5342426 | C | T | T | T | C | T | C | T |
| chr11 | 5345128 | A | G | G | G | A | G | A | G |
| chr11 | 5345170 | G | A | A | A | G | A | G | A |
| chr11 | 5347875 | G | A | A | A | G | A | G | A |
| chr11 | 5347950 | A | A | A | G | A | A | A | G |
| chr11 | 5360615 | G | A | A | A | G | A | G | A |
| chr11 | 5360677 | G | C | C | C | G | C | G | C |
| chr11 | 5364742 | T | C | C | C | T | C | T | C |
| chr11 | 5364776 | TGTC | T | T | T | TGTC | T | TGTC | T |
| chr11 | 5373114 | G | A | G | A | G | G | G | A |
| chr11 | 5373646 | T | C | T | C | T | T | T | C |
| chr11 | 5373713 | T | A | A | A | T | A | T | A |

* Red font mark represents the Gγ(Aγδβ)0 deletion; Highlight text indicates the ‘Codons 41/42(-TTCT)’ mutation. Paternal haplotype with ‘Codons 41/42(-TTCT)’ mutation was marked with blue background; Maternal haplotype with Gγ(Aγδβ)0 mutation was marked with green background.

Table S9* Haplotype of *HBA* gene in family 2

| **Chromosome No.** | **SNP site** | **Genotype of Wife 2** | | **Genotype of Husband 2** | | **Genotype of Offspring 2-1** | | **Genotype of Offspring 2-2** | |
| --- | --- | --- | --- | --- | --- | --- | --- | --- | --- |
| / | / | M1 | M0 | F0 | F1 | M0 | F0 | M0 | F1 |
| chr16 | 91309 | C | T | C | C | T | C | T | C |
| chr16 | 96930 | A | G | G | G | G | G | G | G |
| chr16 | 113493 | C | G | C | C | G | C | G | C |
| chr16 | 115507 | A | G | G | G | G | G | G | G |
| chr16 | 115657 | A | C | A | A | C | A | C | A |
| chr16 | 124140 | G | A | G | G | A | G | / | / |
| chr16 | 124161 | C | G | C | C | G | C | / | / |
| chr16 | 125342 | G | C | G | G | C | G | / | / |
| chr16 | 128991 | CCTGT | C | C | C | C | C | / | / |
| chr16 | 132510 | G | A | A | A | A | A | / | / |
| chr16 | 138019 | T | C | T | T | C | T | C | T |
| chr16 | 138032 | T | C | T | T | C | T | C | T |
| chr16 | 159655 | G | G | C | G | G | C | G | G |
| chr16 | 160001 | A | G | G | G | G | G | / | / |
| chr16 | 176841 | A | G | A | A | G | A | G | A |
| chr16 | 198069 | G | G | T | G | G | T | G | G |
| chr16 | 211167 | G | G | A | G | G | A | G | G |
| chr16 | 221057 | T | C | del | T | C | del | / | / |
| chr16 | 221126 | C | T |  | C | T |  | / | / |
| chr16 | 223597 | T | **C** |  | T | **C** |  | **C** | T |
| chr16 | 224619 | T | C |  | T | C |  | C | T |
| chr16 | 234632 | G | C |  | G | C |  | C | G |
| chr16 | 234710 | T | C |  | T | C |  | C | T |
| chr16 | 234791 | C | CA |  | C | CA |  | CA | C |
| chr16 | 235579 | C | T | C | C | T | C | T | C |
| chr16 | 242770 | A | G | A | A | G | A | G | A |
| chr16 | 259345 | T | G | G | G | G | G | G | G |
| chr16 | 264642 | C | T | T | T | T | T | T | T |
| chr16 | 277458 | A | G | G | A | G | G | G | A |
| chr16 | 300641 | G | A | A | G | A | A | A | G |
| chr16 | 300696 | T | A | A | T | A | A | A | T |
| chr16 | 305377 | G | C | C | G | C | C | C | G |
| chr16 | 312253 | A | G | G | A | G | G | G | A |
| chr16 | 316890 | T | C | C | T | C | C | C | T |
| chr16 | 358736 | A | C | A | A | C | A | C | A |
| chr16 | 358773 | G | A | G | G | A | G | A | G |
| chr16 | 362638 | T | G | T | T | G | T | G | T |
| chr16 | 365024 | C | A | C | C | A | C | A | C |
| chr16 | 369413 | G | A | G | G | A | G | A | G |
| chr16 | 374617 | C | T | C | C | T | C | T | C |
| chr16 | 377623 | A | G | G | A | G | G | G | A |
| chr16 | 392485 | G | A | G | G | A | G | A | G |
| chr16 | 405866 | C | T | C | C | T | C | T | C |
| chr16 | 411092 | T | C | T | T | C | T | C | T |
| chr16 | 413747 | A | G | G | A | G | G | G | A |
| chr16 | 416663 | C | G | G | C | G | G | G | C |
| chr16 | 418429 | A | G | A | A | G | A | G | A |
| chr16 | 418462 | C | G | G | C | G | G | G | C |
| chr16 | 419092 | A | G | G | G | G | G | G | G |
| chr16 | 439550 | T | C | G | T | C | C | C | T |
| chr16 | 439618 | T | T | T | G | T | T | T | G |
| chr16 | 489603 | A | C | C | C | C | C | C | C |
| chr16 | 570623 | A | A | G | A | A | G | A | A |
| chr16 | 622234 | T | T | T | A | T | T | T | A |
| chr16 | 623103 | T | T | T | C | T | T | T | C |
| chr16 | 624114 | A | A | A | G | A | A | A | G |
| chr16 | 625384 | T | T | T | C | T | T | T | C |
| chr16 | 625388 | C | C | C | T | C | C | C | T |
| chr16 | 627920 | T | T | T | C | T | T | T | C |
| chr16 | 630025 | C | C | C | G | C | C | C | G |
| chr16 | 632180 | G | G | G | C | G | G | G | C |
| chr16 | 632198 | T | T | T | A | T | T | T | A |
| chr16 | 632225 | T | T | T | C | T | T | T | C |
| chr16 | 632230 | CG | CG | CG | C | CG | CG | CG | C |
| chr16 | 632233 | T | T | T | C | T | T | T | C |
| chr16 | 632699 | C | C | C | G | C | C | C | G |
| chr16 | 632728 | T | T | T | C | T | T | T | C |
| chr16 | 632736 | T | T | T | C | T | T | T | C |
| chr16 | 632767 | A | G | A | G | A | A | G | G |
| chr16 | 633125 | T | T | T | C | T | T | T | C |
| chr16 | 633353 | TG | TG | TG | CA | TG | TG | TG | CA |
| chr16 | 646421 | A | A | A | G | A | A | A | G |

* Red font mark represents the --SEA deletion; Highlight text indicates the αCS mutation. Paternal haplotype with --SEA mutation was marked with blue background; Maternal haplotype with αCS mutation was marked with green background. ‘/’ represents missing site.

Table S10* Haplotype of *HBB* gene in family 3

| **Chromosome No.** | **SNP site** | **Genotype of Wife 3** | | **Genotype of Husband 3** | | **Genotype of Offspring 3-1** | | **Genotype of Offspring 3-2** | |
| --- | --- | --- | --- | --- | --- | --- | --- | --- | --- |
| / | / | M1 | M0 | F1 | F0 | M0 | F0 | M1 | F0 |
| chr11 | 4937138 | T | C | T | T | C | T | T | T |
| chr11 | 4941587 | C | T | C | C | T | C | C | C |
| chr11 | 4945196 | G | T | G | G | T | G | G | G |
| chr11 | 4992100 | T | T | G | T | T | T | T | T |
| chr11 | 4997634 | A | A | G | A | A | A | A | A |
| chr11 | 5000043 | T | T | C | T | T | T | T | T |
| chr11 | 5006300 | T | T | G | T | T | T | T | T |
| chr11 | 5006406 | TAGTC | TAGTC | T | TAGTC | TAGTC | TAGTC | / | / |
| chr11 | 5031097 | C | C | G | C | C | C | / | / |
| chr11 | 5034741 | A | A | C | A | A | A | / | / |
| chr11 | 5037488 | G | G | A | G | G | G | G | G |
| chr11 | 5039076 | T | T | TGA | T | T | T | / | / |
| chr11 | 5046138 | A | A | G | A | A | A | / | / |
| chr11 | 5051729 | T | T | C | T | T | T | / | / |
| chr11 | 5214597 | T | TTCATC | T | del | TTCATC | del | T | del |
| chr11 | 5214621 | T | C | T |  | C |  | T |  |
| chr11 | 5220001 | C | T | T |  | T |  | C |  |
| chr11 | 5221825 | A | G | G |  | G |  | A |  |
| chr11 | 5222379 | A | G | G |  | G |  | A |  |
| chr11 | 5232573 | G | A | A |  | A |  | G |  |
| chr11 | 5236851 | T | C | C |  | C |  | T |  |
| chr11 | 5247791 | G | C | C |  | C |  | G |  |
| chr11 | 5247992 | CAAAG | **C** | CAAAG |  | **C** |  |  |  |
| chr11 | 5248243 | G | A | A |  | A |  | G |  |
| chr11 | 5249004 | G | A | A |  | A |  | G |  |
| chr11 | 5294145 | C | A | A | A | A | A | C | A |
| chr11 | 5308728 | C | T | T | T | T | T | C | T |
| chr11 | 5310150 | T | C | C | C | C | C | T | C |
| chr11 | 5310169 | A | G | G | G | G | G | A | G |
| chr11 | 5310506 | A | C | C | C | C | C | A | C |
| chr11 | 5310578 | G | A | A | A | A | A | G | A |
| chr11 | 5311063 | A | T | T | T | T | T | A | T |
| chr11 | 5312401 | A | G | G | G | G | G | A | G |
| chr11 | 5331644 | A | A | A | G | A | G | A | G |
| chr11 | 5342404 | T | C | C | C | C | C | T | C |
| chr11 | 5342426 | T | T | T | C | T | C | T | C |
| chr11 | 5345128 | G | G | G | A | G | A | G | A |
| chr11 | 5347875 | A | A | A | G | A | G | A | G |
| chr11 | 5360615 | A | A | A | G | A | G | A | G |
| chr11 | 5360677 | C | C | C | G | C | G | C | G |
| chr11 | 5373713 | A | A | A | T | A | T | A | T |

* Red font mark represents the Gγ(Aγδβ)0 deletion; Highlight text indicates the ‘Codons 41/42(-TTCT)’ mutation. Maternal haplotype with ‘Codons 41/42(-TTCT)’ mutation was marked with green background; Paternal haplotype with Gγ(Aγδβ)0 mutation was marked with blue background. ‘/’ represents missing site.

Table S11* Haplotype of *HBB* gene in family 4.

| **Chromosome No.** | **SNP site** | **Genotype of Wife 4** | | **Genotype of Husband 4** | | **Genotype of Offspring 4-1** | | **Genotype of Offspring 4-2** | |
| --- | --- | --- | --- | --- | --- | --- | --- | --- | --- |
| / | / | M0 | M1 | F1 | F0 | M0 | F0 | M1 | F0 |
| chr11 | 4908907 | G | A | G | G | G | G | A | G |
| chr11 | 4909000 | G | A | G | G | G | G | A | G |
| chr11 | 4925167 | A | C | A | A | A | A | C | A |
| chr11 | 4928866 | C | T | C | C | C | C | T | C |
| chr11 | 4933497 | A | AAATAATTT | A | A | A | A | AAATAATTT | A |
| chr11 | 4937040 | G | T | T | T | G | T | T | T |
| chr11 | 4937319 | C | T | T | T | C | T | T | T |
| chr11 | 5026200 | C | C | T | C | C | C | C | C |
| chr11 | 5031097 | C | G | G | C | C | C | G | C |
| chr11 | 5034662 | A | T | T | A | A | A | T | A |
| chr11 | 5034741 | A | C | C | A | A | A | C | A |
| chr11 | 5042941 | C | T | T | C | C | C | T | C |
| chr11 | 5046010 | G | A | G | G | G | G | A | G |
| chr11 | 5051778 | G | A | G | G | G | G | A | G |
| chr11 | 5053790 | T | C | C | T | T | T | C | T |
| chr11 | 5058271 | C | T | T | C | C | C | T | C |
| chr11 | 5059420 | T | C | C | T | T | T | C | T |
| chr11 | 5059443 | T | C | C | T | T | T | C | T |
| chr11 | 5061996 | T | C | C | T | T | T | C | T |
| chr11 | 5062022 | C | T | T | C | C | C | T | C |
| chr11 | 5062029 | A | G | A | A | A | A | G | A |
| chr11 | 5062840 | G | A | G | G | G | G | A | G |
| chr11 | 5062859 | A | G | G | A | A | A | G | A |
| chr11 | 5062889 | G | C | C | G | G | G | C | G |
| chr11 | 5068137 | G | A | A | G | G | G | A | G |
| chr11 | 5068177 | A | T | T | A | A | A | T | A |
| chr11 | 5069793 | C | T | T | C | C | C | T | C |
| chr11 | 5069834 | G | A | A | G | G | G | A | G |
| chr11 | 5072557 | G | T | T | G | G | G | T | G |
| chr11 | 5072581 | TG | CA | CA | TG | TG | TG | CA | TG |
| chr11 | 5072621 | T | G | G | T | T | T | G | T |
| chr11 | 5072622 | T | TAA | TAA | T | T | T | TAA | T |
| chr11 | 5072638 | T | C | C | T | T | T | C | T |
| chr11 | 5072707 | C | T |  | C | C | C | T | C |
| chr11 | 5115238 | T | C | C | C | T | C | C | C |
| chr11 | 5115874 | T | C | C | C | T | C | C | C |
| chr11 | 5118880 | G | A | A | A | G | A | A | A |
| chr11 | 5118949 | A | G | G | G | A | G | G | G |
| chr11 | 5125396 | C | A | A | A | C | A | A | A |
| chr11 | 5128411 | A | G | G | G | A | G | G | G |
| chr11 | 5128423 | T | C | C | C | T | C | C | C |
| chr11 | 5132625 | G | A | A | A | G | A | A | A |
| chr11 | 5141876 | G | A | A | A | G | A | A | A |
| chr11 | 5141902 | T | C | C | C | T | C | C | C |
| chr11 | 5194616 | C | G | G | G | C | G | G | G |
| chr11 | 5214597 | TTCATC | T | TTCATC | TTCATC | TTCATC | TTCATC | T | TTCATC |
| chr11 | 5214621 | T | T | C | T | T | T | T | T |
| chr11 | 5218808 | A | A | AAT | A | A | A | A | A |
| chr11 | 5221825 | A | G | A | A | A | A | G | A |
| chr11 | 5222340 | A | G | G | A | A | A | G | A |
| chr11 | 5222379 | G | G | A | A | G | A | G | A |
| chr11 | 5222399 | A | G | G | A | A | A | G | A |
| chr11 | 5246203 | C | A | A | C | C | C | A | C |
| chr11 | **5247153** | **T** | C | C | **T** | **T** | **T** | C | **T** |
| chr11 | 5247733 | C | A | A | C | C | C | A | C |
| chr11 | 5247791 | G | C | G | G | G | G | C | G |
| chr11 | 5248243 | G | A | G | G | G | G | A | G |
| chr11 | 5272154 | C | T | C | C | C | C | T | C |
| chr11 | 5273147 | G | C | G | G | G | G | C | G |
| chr11 | 5273541 | A | G | A | A | A | A | G | A |
| chr11 | 5273687 | G | T | G | G | G | G | T | G |
| chr11 | 5280022 | C | G | C | C | C | C | G | C |
| chr11 | 5284978 | G | A | G | G | G | G | A | G |
| chr11 | 5286312 | C | T | C | C | C | C | T | C |
| chr11 | 5286808 | C | T | C | C | C | C | T | C |
| chr11 | 5287935 | T | C | T | T | T | T | C | T |
| chr11 | 5308728 | T | C | T | T | T | T | C | T |
| chr11 | 5310150 | C | T | C | C | C | C | T | C |
| chr11 | 5310169 | G | A | G | G | G | G | A | G |
| chr11 | 5310506 | C | A | C | C | C | C | A | C |
| chr11 | 5310578 | A | G | A | A | A | A | G | A |
| chr11 | 5311063 | T | A | T | T | T | T | A | T |
| chr11 | 5312401 | G | A | G | G | G | G | A | G |
| chr11 | 5370509 | T | T | T | A | T | A | T | A |
| chr11 | 5373114 | G | G | G | A | G | A | G | A |
| chr11 | 5373646 | T | T | T | C | T | C | T | C |

* Red font mark represents the IVS-II-654 (C>T) mutation site. Maternal haplotype with IVS-II-654 (C>T) mutation was marked with green background; Paternal haplotype with IVS-II-654 (C>T) mutation was marked with blue background.

**2.4 Lab 4**


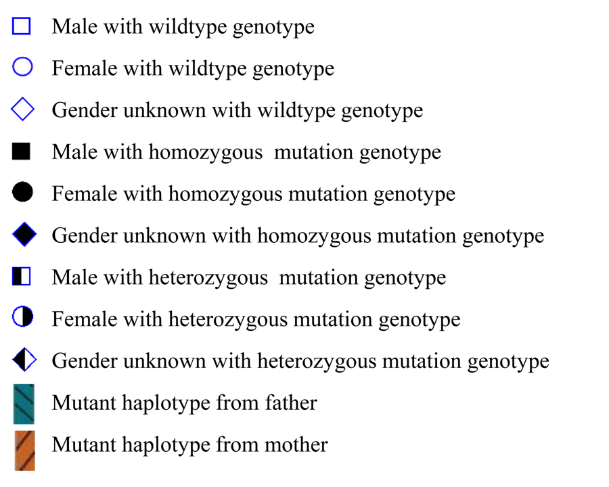

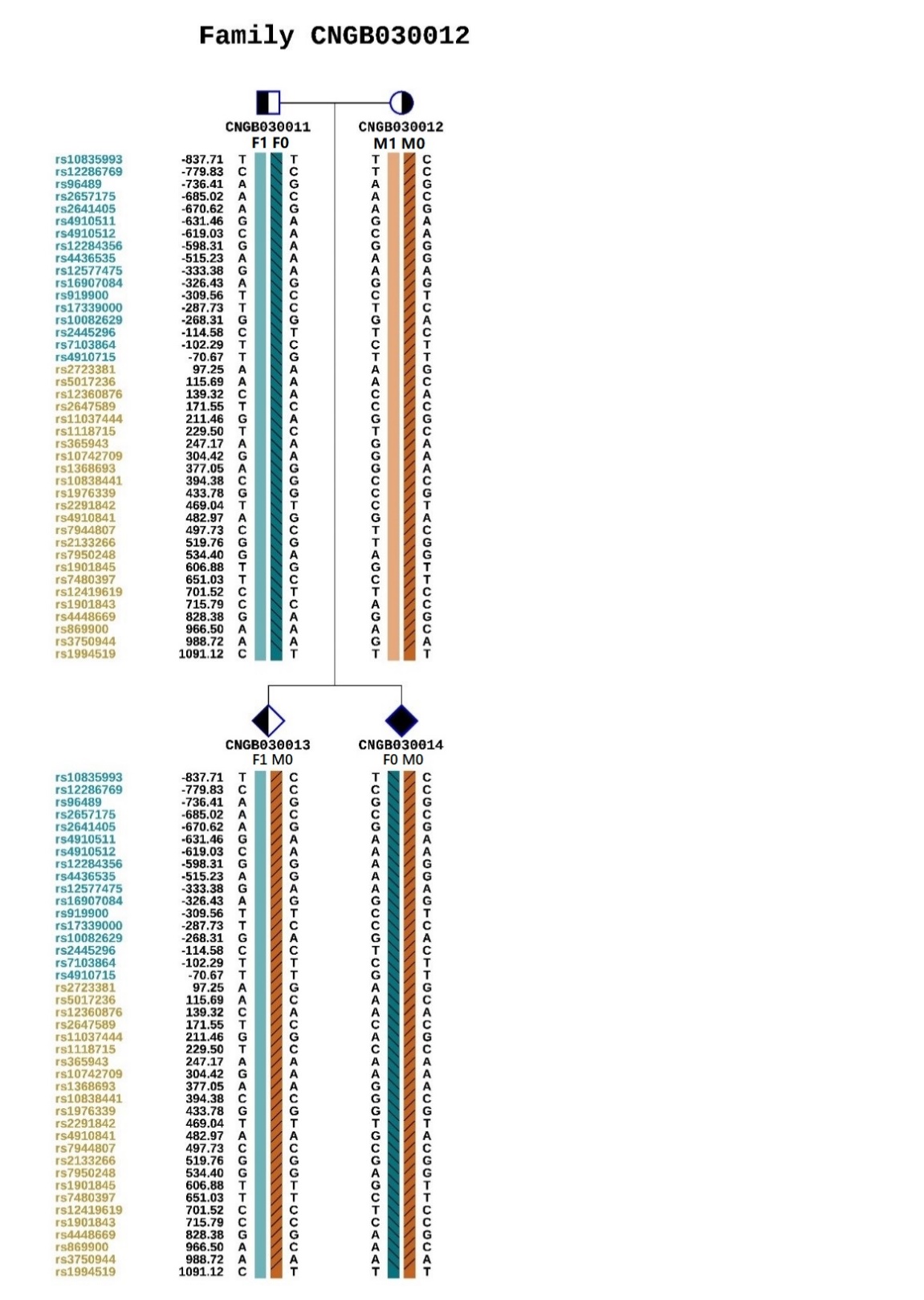


Figure S1 Haplotype phasing of family 1 in Lab 4. Left, Haplotype phasing of *HBB* gene. Black font numbers indicate the distance (Kb) upstream (-) or downstream (+) of mutant region. Blue and orange number indicate the SNP sites in the chromosome.


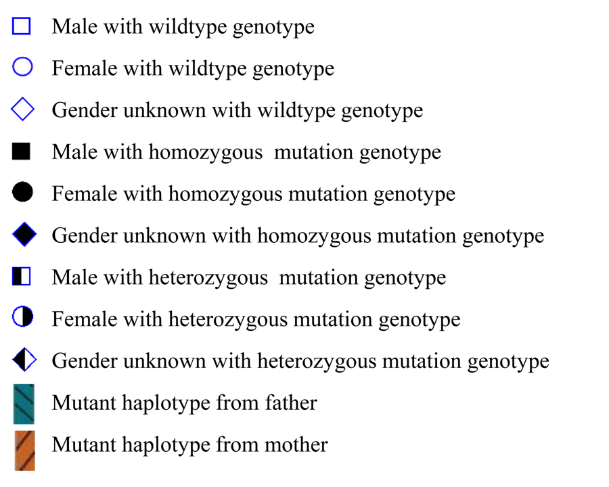

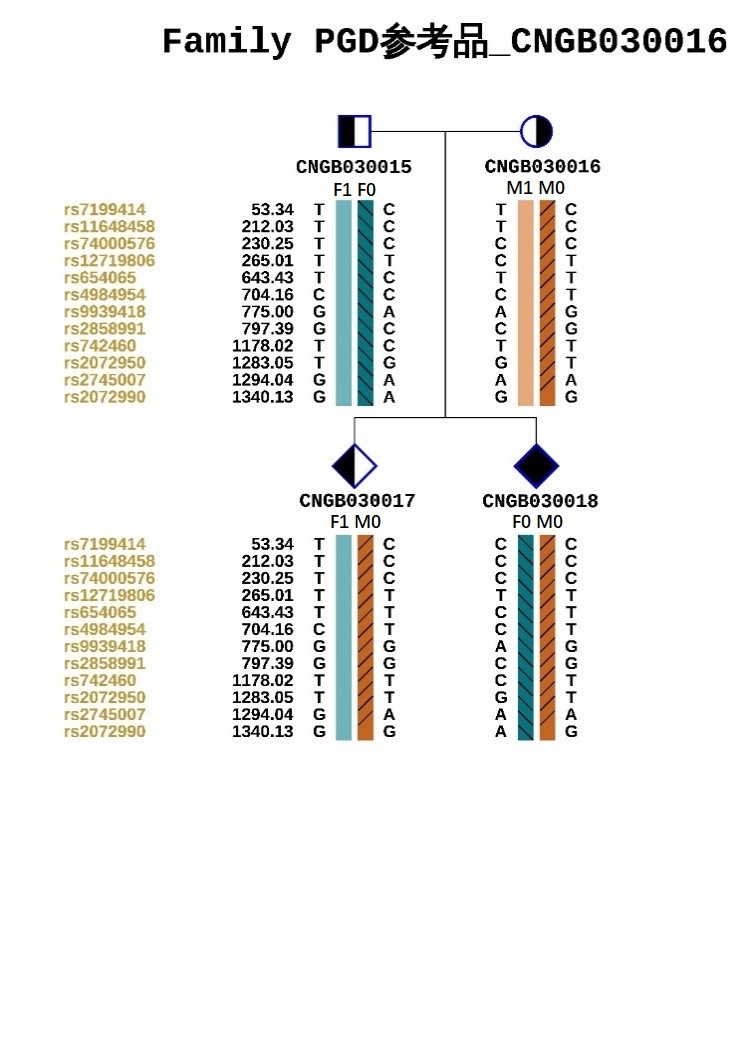


Figure S2 Haplotype phasing of family 2 in Lab 4. Haplotype phasing of *HBA* gene. Black font numbers indicate the distance (Kb) downstream (+) of mutant region. Blue number indicate the SNP sites in the chromosome.


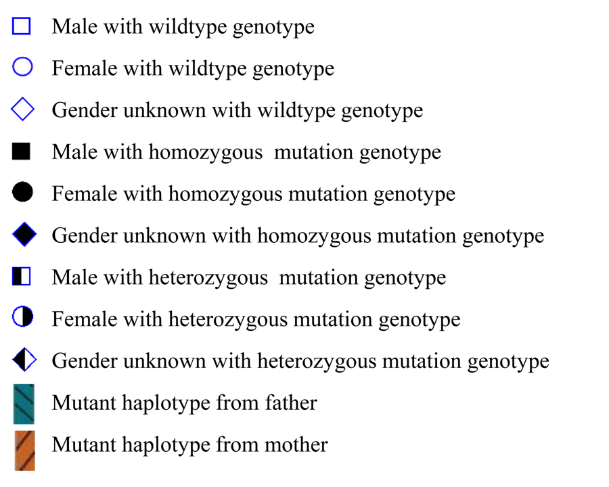

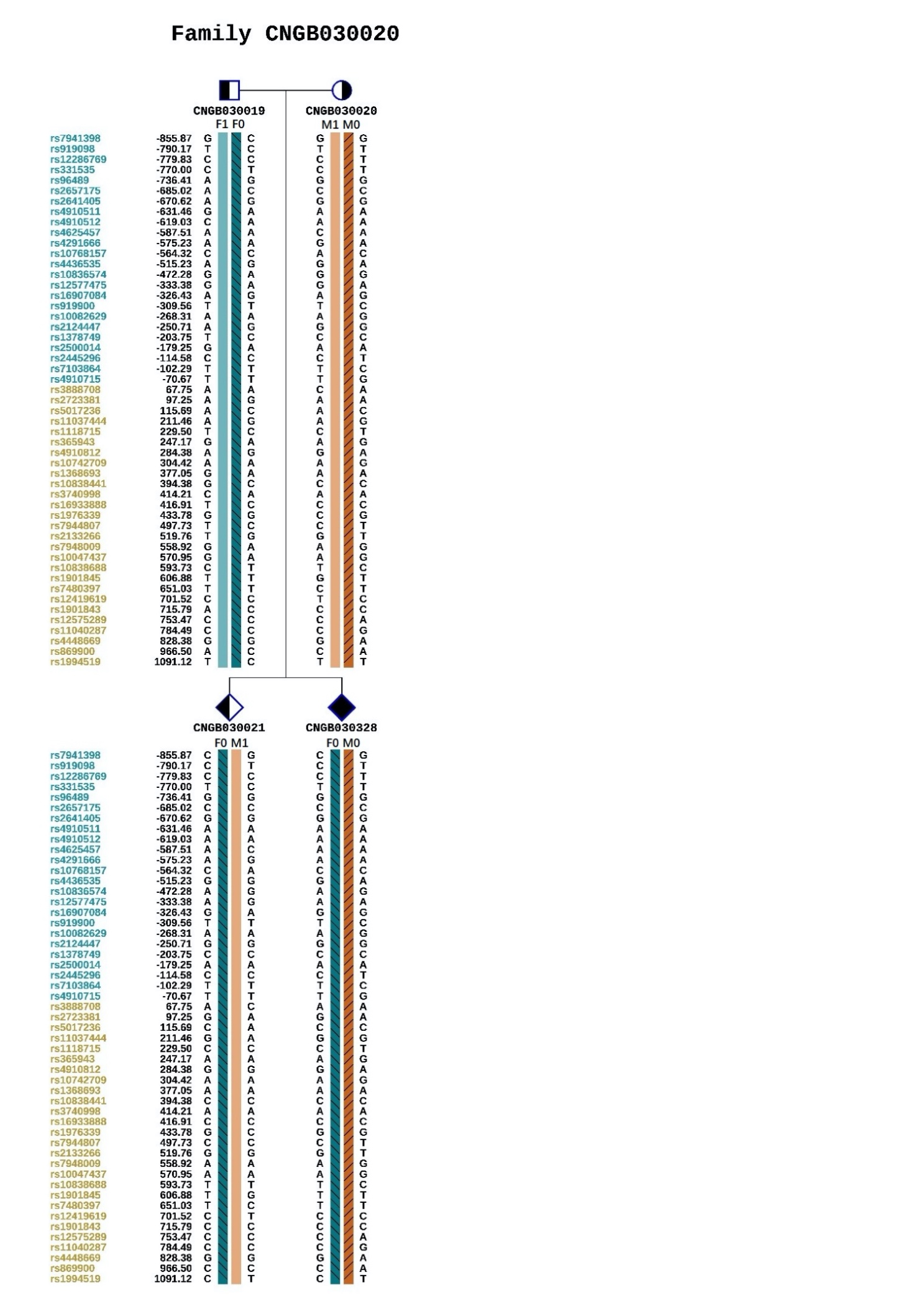


Figure S3 Haplotype phasing of family 3 in Lab 4. Haplotype phasing of *HBB* gene. Black font numbers indicate the distance (Kb) upstream (-) or downstream (+) of mutant region. Blue and orange number indicate the SNP sites in the chromosome.


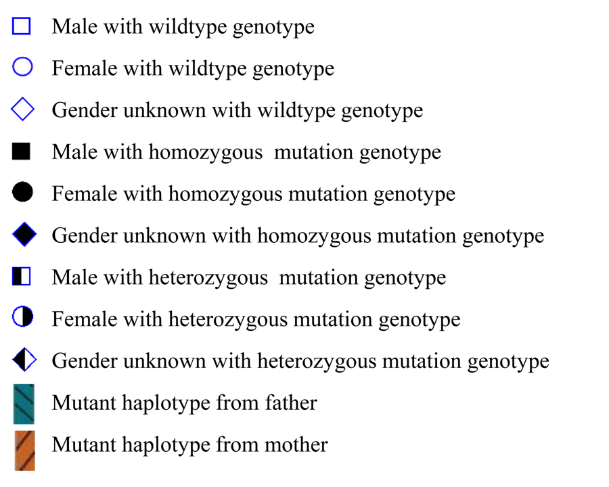

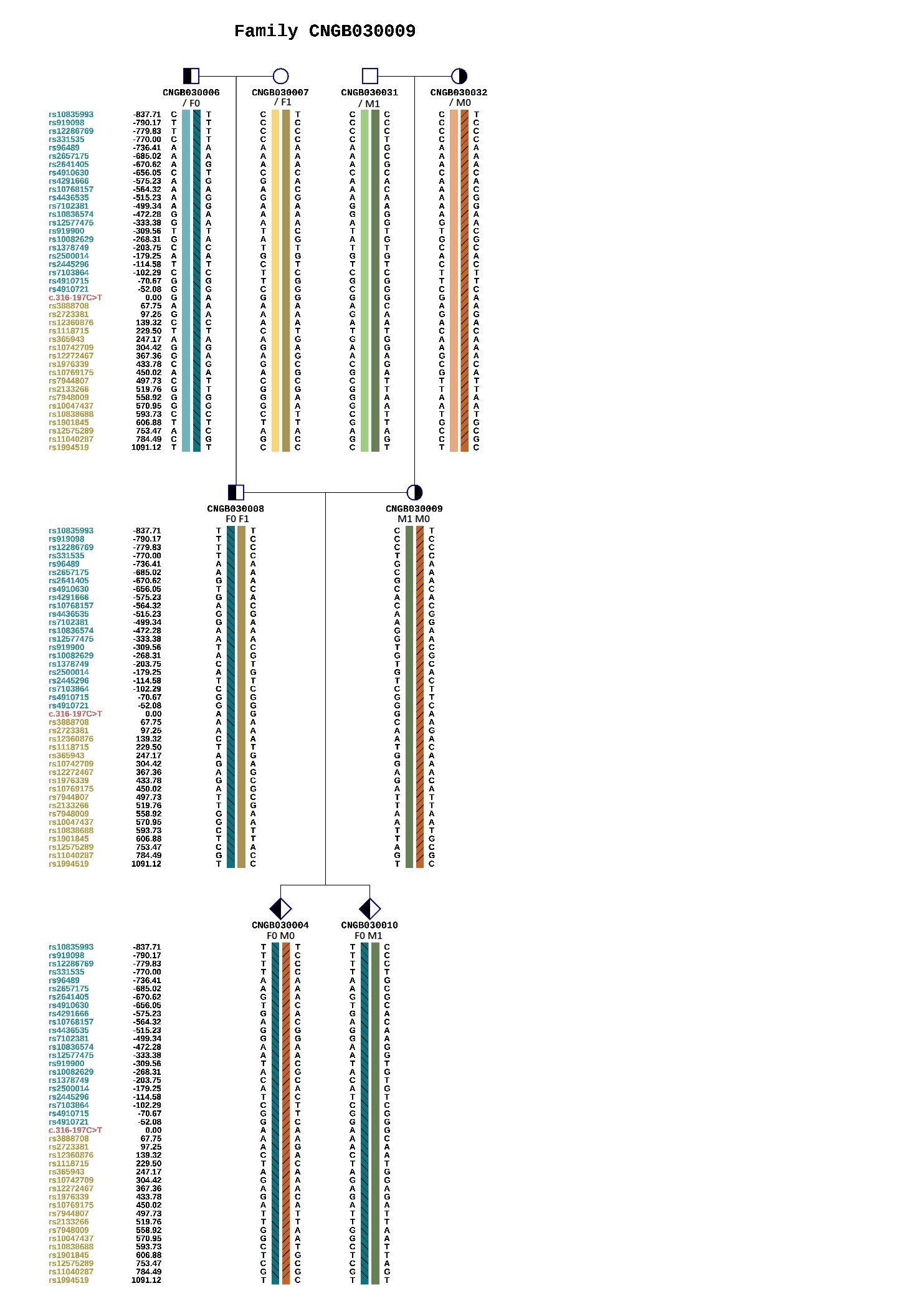


Figure S4 Haplotype phasing of family 4 in Lab 4. Haplotype phasing of *HBB* gene. Black font numbers indicate the distance (Kb) upstream (-) or downstream (+) of mutant region. Blue and orange number indicate the SNP sites in the chromosome.

**3 Stability tests of gDNA samples and cell samples**


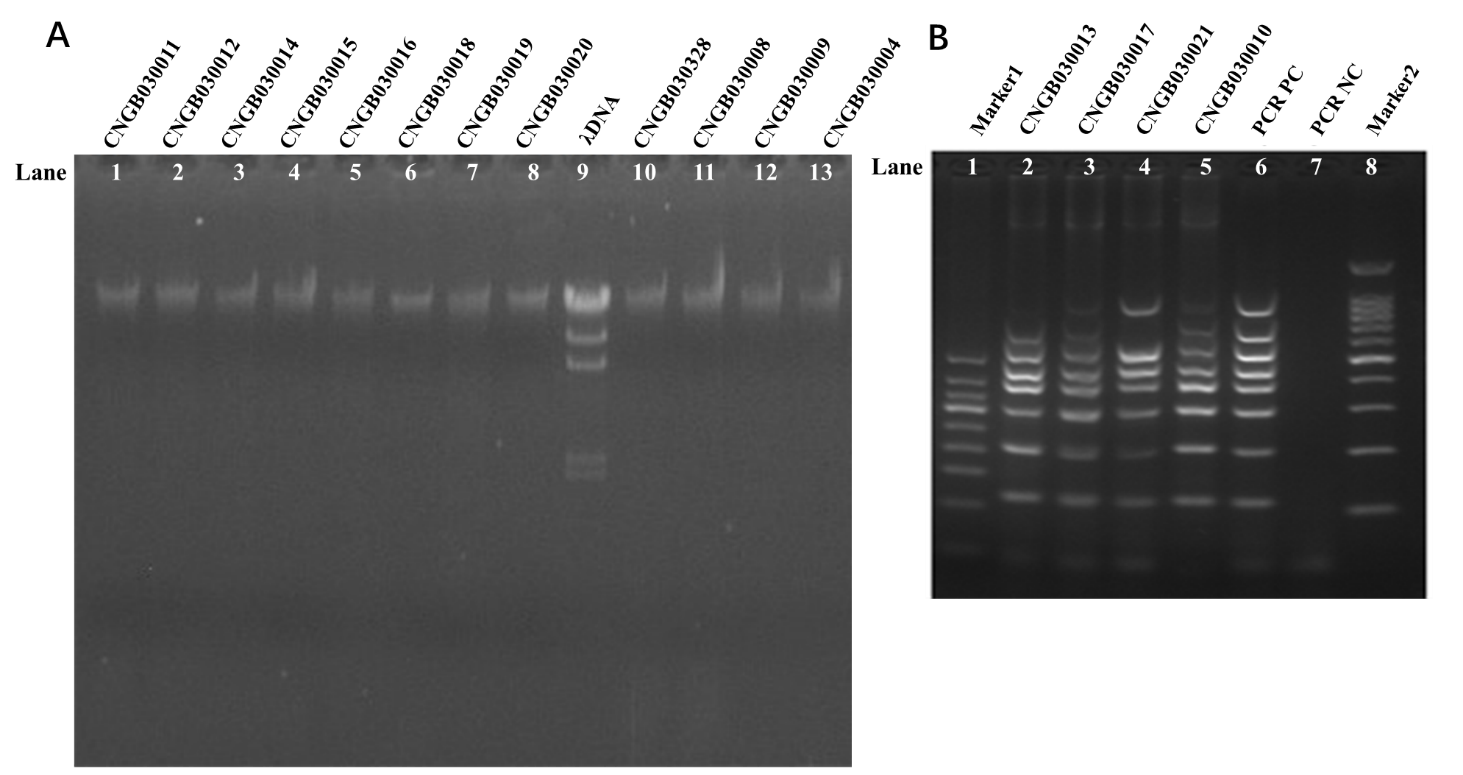


Figure S5. A) Freeze-thawing test of the gDNA and quality control of the cellular samples. gDNA samples after 6 repeated freeze-thawing cycles were subjected to 2% agarose gel electrophoresis, phage λDNA/Hind III digest was used as marker. B) Quick quality control of the cell samples after cell sorting. Eight amplicons of housekeeping genes were separated by 2% agarose gel. PCR PC: NA12878, PCR NC: H2O, Marker1: 500, 400, 350, 300 (enhanced bands), 250, 100, 150, 100, 50 bp; Marker 2: 1500, 1000, 900, 800, 700, 600, 500 (enhanced bands), 400, 300, 200, 100 bp.


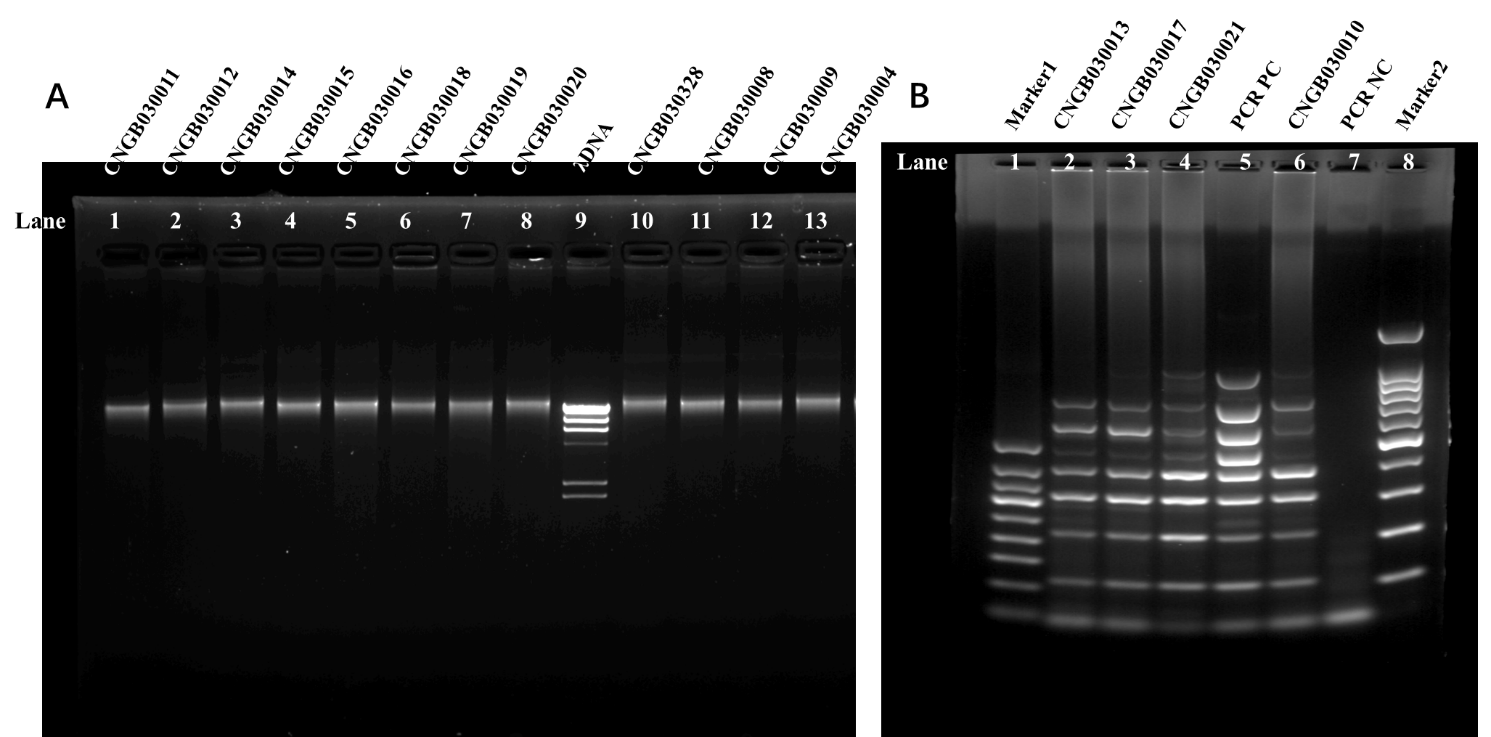


Figure S6. Evaluating the storage stability of the PGT-M RMs for thalassemia. A) gDNA samples after 3 years of storage were subjected to 2% agarose gel electrophoresis, phage λDNA/Hind III digest was used as marker. B) cellular samples after 3 years of storage were subjected to MDA and housekeeping genes amplification. Eight amplicons of housekeeping genes were separated by 2% agarose gel. PCR PC: NA12878, PCR NC: H2O, Marker1: 500, 400, 350, 300 (enhanced bands), 250, 100, 150, 100, 50 bp; Marker 2: 1500, 1000, 900, 800, 700, 600, 500 (enhanced bands), 400, 300, 200, 100 bp.
